# Intestinal protection by proanthocyanidins involves anti-oxidative and anti-inflammatory actions in association with an improvement of insulin sensitivity, lipid and glucose homeostasis

**DOI:** 10.1038/s41598-020-80587-5

**Published:** 2021-02-16

**Authors:** Mireille Koudoufio, Francis Feldman, Lena Ahmarani, Edgard Delvin, Schohraya Spahis, Yves Desjardins, Emile Levy

**Affiliations:** 1 Research Centre, CHU Sainte-Justine, Université de Montréal, 3175 Ste Catherine Road, Montreal, QC H3T 1C5 Canada; 2 Department of Nutrition, Université de Montréal, Montreal, QC H3T 1A8 Canada; 3 Department of Biochemistry, Université de Montréal, Montreal, QC H3C 3J7 Canada; 4 Institute of Nutrition and Functional Foods (INAF), Université Laval, Quebec, QC G1V 0A6 Canada

**Keywords:** Cell biology, Biomarkers, Diseases

## Abstract

Recent advances have added another dimension to the complexity of cardiometabolic disorders (CMD) by directly implicating the gastrointestinal tract as a key player. In fact, multiple factors could interfere with intestinal homeostasis and elicit extra-intestinal CMD. As oxidative stress (OxS), inflammation, insulin resistance and lipid abnormalities are among the most disruptive events, the aim of the present study is to explore whether proanthocyanidins (PACs) exert protective effects against these disorders. To this end, fully differentiated intestinal Caco-2/15 cells were pre-incubated with PACs with and without the pro-oxidant and pro-inflammatory iron/ascorbate (Fe/Asc). PACs significantly reduce malondialdehyde, a biomarker of lipid peroxidation, and raise antioxidant SOD2 and GPx via the increase of NRF2/Keap1 ratio. Likewise, PACs decrease the inflammatory agents TNFα and COX2 through abrogation of NF-κB. Moreover, according to crucial biomarkers, PACs result in lipid homeostasis improvement as reflected by enhanced fatty acid β-oxidation, diminished lipogenesis, and lowered gluconeogenesis as a result of PPARα, γ and SREBP1c modulation. Since these metabolic routes are mainly regulated by insulin sensitivity, we have examined the insulin signaling pathway and found an upregulation of phosphoPI3K/Akt and downregulation of p38-MAPK expressions, indicating beneficial effects in response to PACs. Taken together, PACs display the potential to counterbalance OxS and inflammation in Fe/Asc-exposed intestinal cells, in association with an improvement of insulin sensitivity, which ameliorates lipid and glucose homeostasis.

## Introduction

The gastrointestinal (GI) tract is a complex system that fulfills essential functions in the human body^1–3^. It ensures digestion and absorption of nutrients, contributes to overall immunology competence by providing protection against pathogens, produces multiple peptidic hormones acting on several tissues and pathways, and harbors microbial communities interacting with the host and influencing metabolic health^4,5^. In addition, the intestinal epithelium protects from foreign pathogen invasion and serves as the first line of defense, which is crucial for the prevention of multiple disorders^6^.

The GI tract is continuously exposed to harmful stimuli that may cause oxidative stress (OxS), inflammation and injury. Intraluminal pro-oxidants from ingested nutrients contain varying concentrations of lipid oxidation products, such as cholesterol oxides^7,8^. The intestinal epithelium is also subject to multiple oxidant assaults from catalase-negative bacteria, oxidase-producing desquamated cells (e.g. xanthine oxidase), and hypothiocyanous acid-containing saliva, which increase reactive oxygen species (ROS) in intestinal lumen^9^. Importantly, ROS are able to damage cell membranes, disturb barrier integrity, and lead to enhanced intestinal permeability, inflammation and endotoxemia^10^. The combination of OxS and inflammation in the gut compromises immune, digestive, endocrine and nervous functions^11^. In particular, it elicits a decline in insulin sensitivity (IS), which triggers metabolic disorders^12,13^.

Recently, growing evidence suggests that polyphenols and derivatives have the potential to prevent and treat chronic diseases^14^. These plant secondary metabolites exhibit various biological features and are gaining a growing interest among scientists. They are capable of reducing ROS generation, neutralize OxS development, and fight inflammation^15,16^. However, the mechanisms of action resulting in beneficial outcomes remain elusive. Furthermore, given their limited absorption and bioavailability, investigations into their physiological functions were limited to polyphenol transport and catabolism in the gut.

The main objective of the study is to determine the modulation of OxS and inflammation by proanthocyanidins (PACs), which are abundant flavan-3-ols in dietary fruits, vegetables, nuts and grains. As OxS and inflammation are two crucial processes promoting IS, insulin signaling is evaluated in response to PACs. The impact of these polyphenols is also assessed on fatty acids (FA) β-oxidation and lipogenesis, which will allow to estimate lipid homeostasis. The mechanisms behind PACs influence are appraised by focusing on central transcription factors. To this end, we have used the well-established human-derived immortalized Caco-2/15 cell line. These intestinal cells are known to undergo a spontaneous cell differentiation leading to the formation of a cell monolayer expressing several morphological and functional characteristics of mature human enterocytes^17–19^.

## Results

### Caco 2/15 cell integrity following different treatments

**Figure 1:**
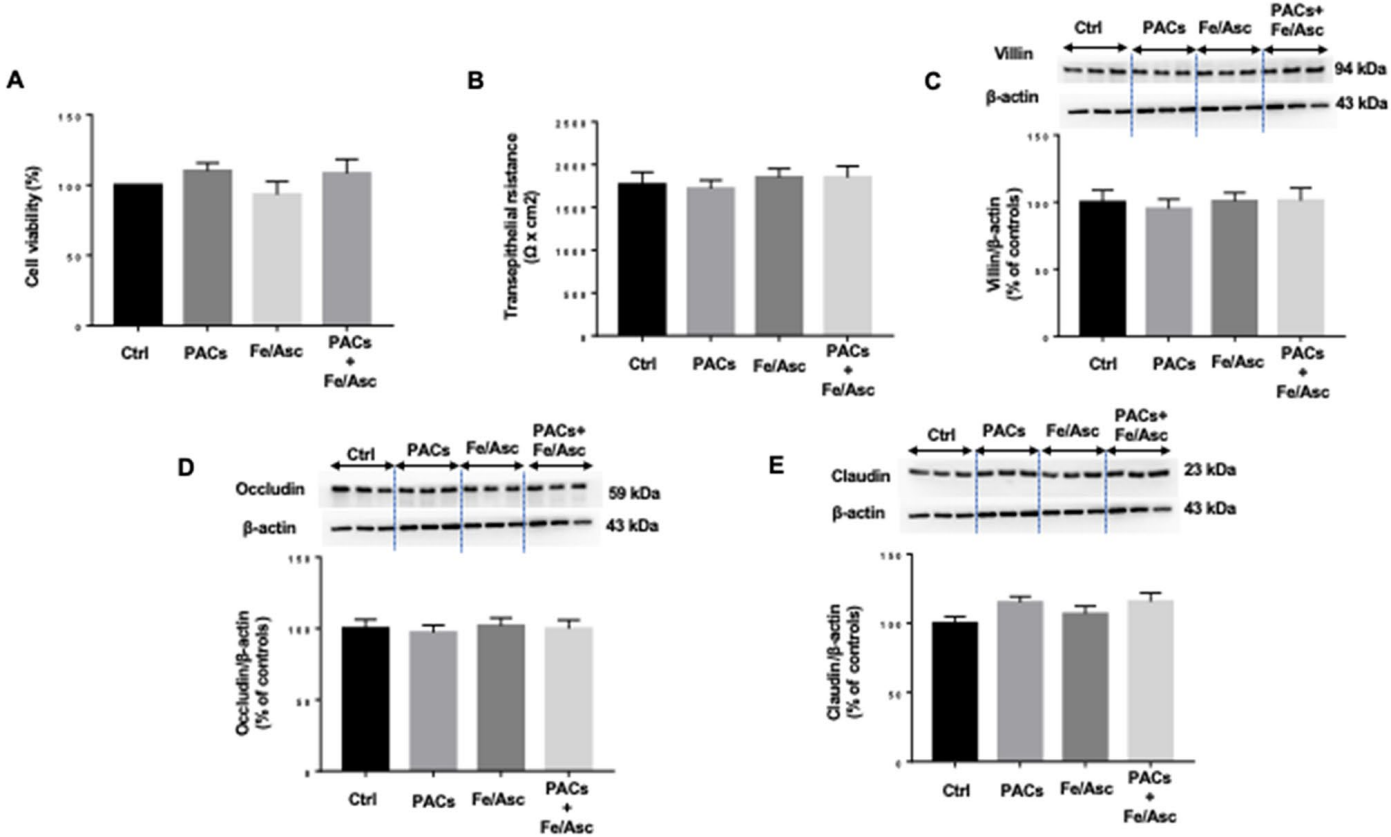
Effects of PACs on cell integrity and viability of Caco2/15 cells. Integrity of Caco 2/15 monolayer was determined by cell viability, differentiation and tight junction assays on fully differentiated state. Proanthocyanidins (PACs, 250 µg/mL) were added to the apical cell compartment for 24 h prior to treatment with Fe/Asc (200 µM/2 mM) for 6 h at 37 °C. (**A**) MTT was used to assay cell viability, and (**B**) transepithelial resistance was also determined. (**C**) Villin, (**D**) Occludin and (**E**) Claudin protein expressions were assessed by Western blot. Results represent the means ± SEM of 3 independent experiments, each in triplicate. A representative Western blot is shown, illustrating an experiment in triplicate on the same gel and at the same exposure.

Before evaluating the impact of polyphenols on various processes, we wanted to ensure that Caco-2/15 cells maintained their integrity in response to iron/ascorbate (Fe/Asc) and PACs treatment. Cell viability was not affected as observed from the invariable 3-(4,5-dimethyldiazol-2-yl)-2,5 diphenyl Tetrazolium Bromid (MTT) assay (Fig. 1A). Confirmation of integrity preservation was obtained following analysis by trypan blue exclusion (data not shown). Furthermore, measurement of transepithelial electrical resistance did not disclose any significant modification of the barrier integrity of Caco-2/15 cells (Fig. 1B). Finally, cell differentiation was not disturbed according to villin, occludin and claudin protein expression (Fig. 1C–E). Therefore, the incubation of Caco-2/15 cells with Fe/Asc (200 μM/2 mM) ± 250 µg/mL PACs did not affect cell viability, integrity and differentiation.

### Effects of PACs on Fe/Asc-induced OxS

**Figure 2:**
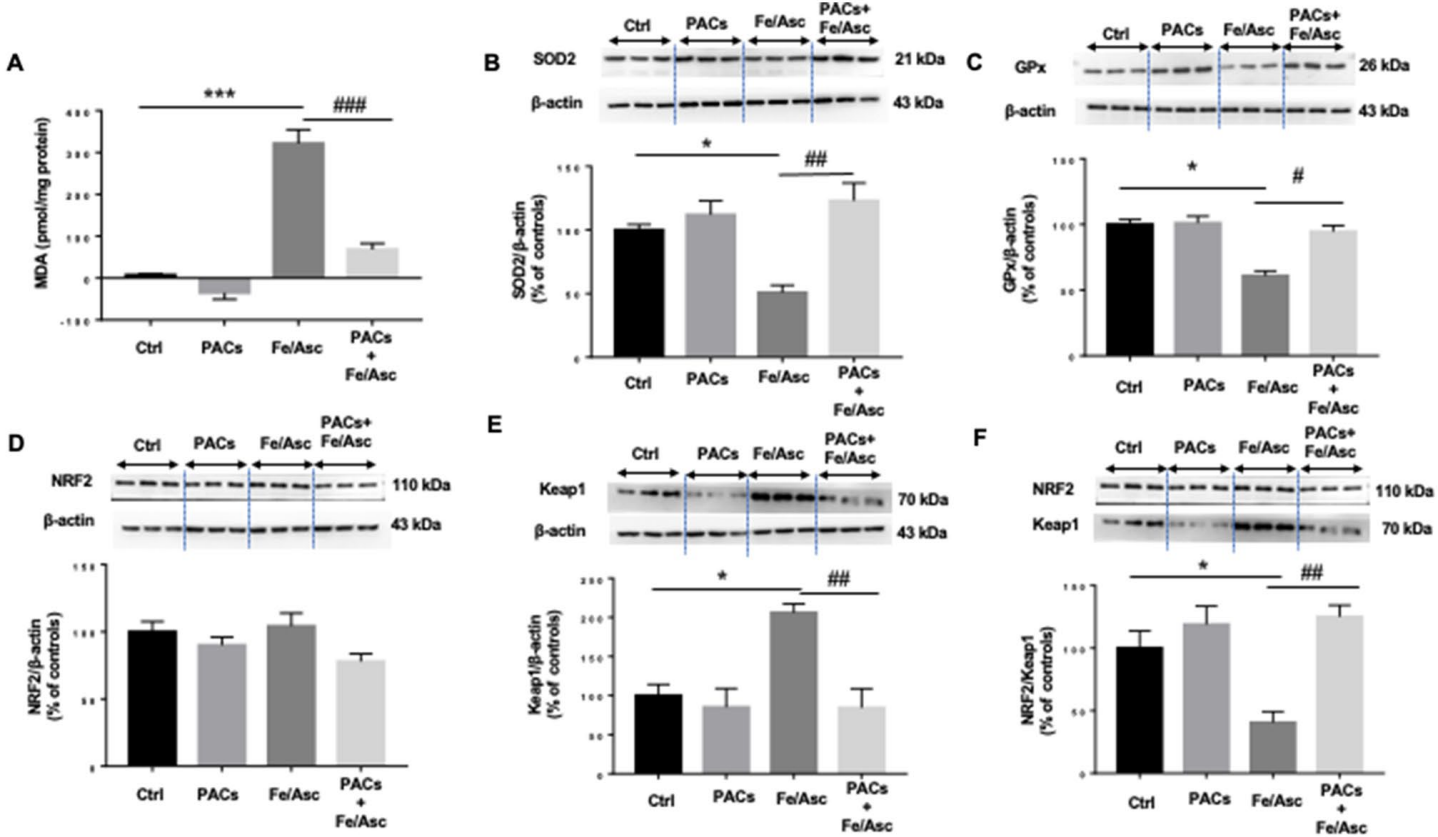
Effects of PACs on lipid peroxidation, endogenous antioxidants, and oxidative stress key transcription factors in Caco-2/15 cells. Proanthocyanidins (PACs, 250 µg/mL) were added to the apical cell compartment for 24 h prior to treatment with Fe/Asc (200 µM/2 mM) for 6 h at 37 °C. Lipid peroxidation was determined by measuring (**A**) MDA levels by HPLC. The protein expression of (**B**) SOD2, (**C**) GPx, (**D**) NRF2 and (**E**) Keap 1 was determined by Western blot. (**F**) The ratio NRF2/Keap1 was calculated. Results represent the means ± SEM of 3 independent experiments, each in triplicate. A representative Western blot is shown, illustrating an experiment in triplicate on the same gel and with the same exposure. *P < 0.05; ***P < 0.001 vs untreated cells (ctrl). ^#^P < 0.05; ^##^P < 0.01; ^###^P < 0.001 vs Fe/Asc-treated cells. *MDA* malondialdehyde, *SOD2* superoxide dismutase 2, *GPx* Glutathione peroxidase, *NRF2* Nuclear factor erythroid 2-related factor 2, *Keap1* Kelch-like ECH-associated protein.

We then estimated the induction of OxS following Fe/Asc administration. Assessment by HPLC of the malondialdehyde (MDA), as a biomarker of lipid peroxidation, showed an elevated MDA content compared with control Caco-2/15 cells (Fig. 2A). Consistently, the protein expression of the antioxidant superoxide dismutase 2 (SOD2) and glutathione peroxidase (GPx) enzymes was reduced by Fe/Asc compared to untreated cells (Fig. 2B,C). Nonetheless, the presence of PACs counteracted Fe/Asc-mediated lipid peroxidation as evidenced by MDA lessening as well as SOD2 and GPx elevation. As the transcription factor nuclear factor erythroid-2-related factor 2 (NRF2) is critical for the regulation of endogenous antioxidants, we evaluated its protein expression (Fig. 2D). No changes in NRF2 were detected in Caco-2/15 cells treated with Fe/Asc and PACs, but the protein expression of its negative regulator Kelch-like ECH-associated protein 1 (Keap1), a biosensor for oxidative and electrophilic stresses, was upregulated by Fe/Asc and downregulated by PACs (Fig. 2E). In particular, the important ratio of NRF2/Keap1, which is an indicator of a transcriptional activation of NRF2, followed the same trend (Figs. 2F). Overall, PACs are effective in counteracting Fe/Asc-mediated OxS.

### PAC effects and mechanisms of actions with respect to inflammatory process

**Figure 3:**
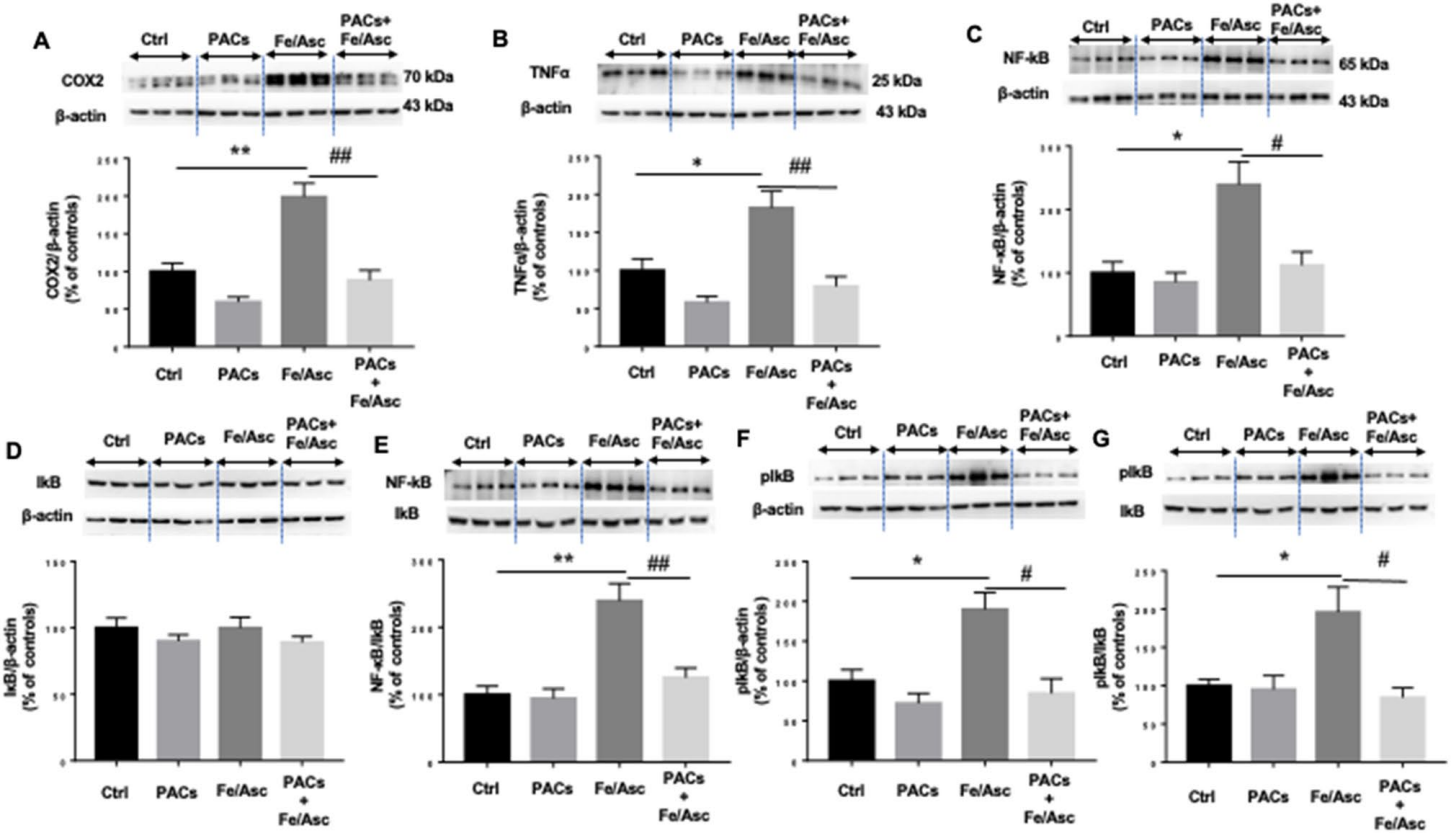
Effects of PACs on inflammatory markers and the key transcription factor NF-kB in Fe/Asc-induced inflammation in Caco-2/15 cells. Proanthocyanidins (PACs, 250 µg/mL) were added to the apical cell compartment for 24 h prior to treatment with Fe/Asc (200 µM/2 mM) for 6 h at 37 °C. Protein expressions of (**A**) COX2 and (**B**) TNFα, (**C**) NF-kB, (**D**) IkB and its (**F**) phosphorylated form (pIkB) were determined by Western blot. The ratios (**E**) NF-kB/IkB and (**G**) pIkB/IkB were calculated. Results represent the means ± SEM of 3 independent experiments, each in triplicate. A representative Western blot is shown, illustrating an experiment in triplicate on the same gel and with the same exposure. *P < 0.05; **P < 0.01 vs untreated cells (ctrl). ^#^P < 0.05; ^##^P < 0.01 vs Fe/Asc treated cells. *COX2* Cyclooxygenase-2, *TNFα* Tumor necrosis factor alpha, *NF-κB* nuclear transcription factor-kappa B, *IκB* Inhibitor of kappa B.

Pro-inflammatory cytokines are triggered in intestinal epithelial cells in OxS conditions. Our experiments confirmed this observation by demonstrating a Fe/Asc-mediated tumor necrosis factor-alpha (TNFα) induction (Fig. 3). In line with these data, the protein expression of cyclooxygenase-2 (COX2), an enzyme exerting multifaceted role in inflammation promotion and perpetuation, was increased by the pro-oxidant Fe/Asc. However, PAC administration offset TNFα and COX2 protein levels elicited by Fe/Asc (Fig. 3A,B). Since nuclear factor kappa-B (NF-κB) represents a powerful mediator of inflammatory responses, we tested its implication in the transcription of the aforementioned inflammatory components (Fig. 3C). Whereas Caco-2/15 cells challenged by Fe/Asc exhibited a high NF-κB signal likely through activating NF-κB/IκB ratio (Fig. 3E) and phosphorylation of its inhibitor kappa B (IκB) (Fig. 3F), PACs were able to interfere with the NF-κB pathway, as reflected by their inhibition of NF-κB and curtailment of IκB phosphorylation/IκB ratio, which was raised by Fe/Asc (Fig. 3G). Therefore, PACs work as a potently repressing agent of inflammation.

### Regulation of intracellular lipid metabolism by PACs

**Figure 4:**
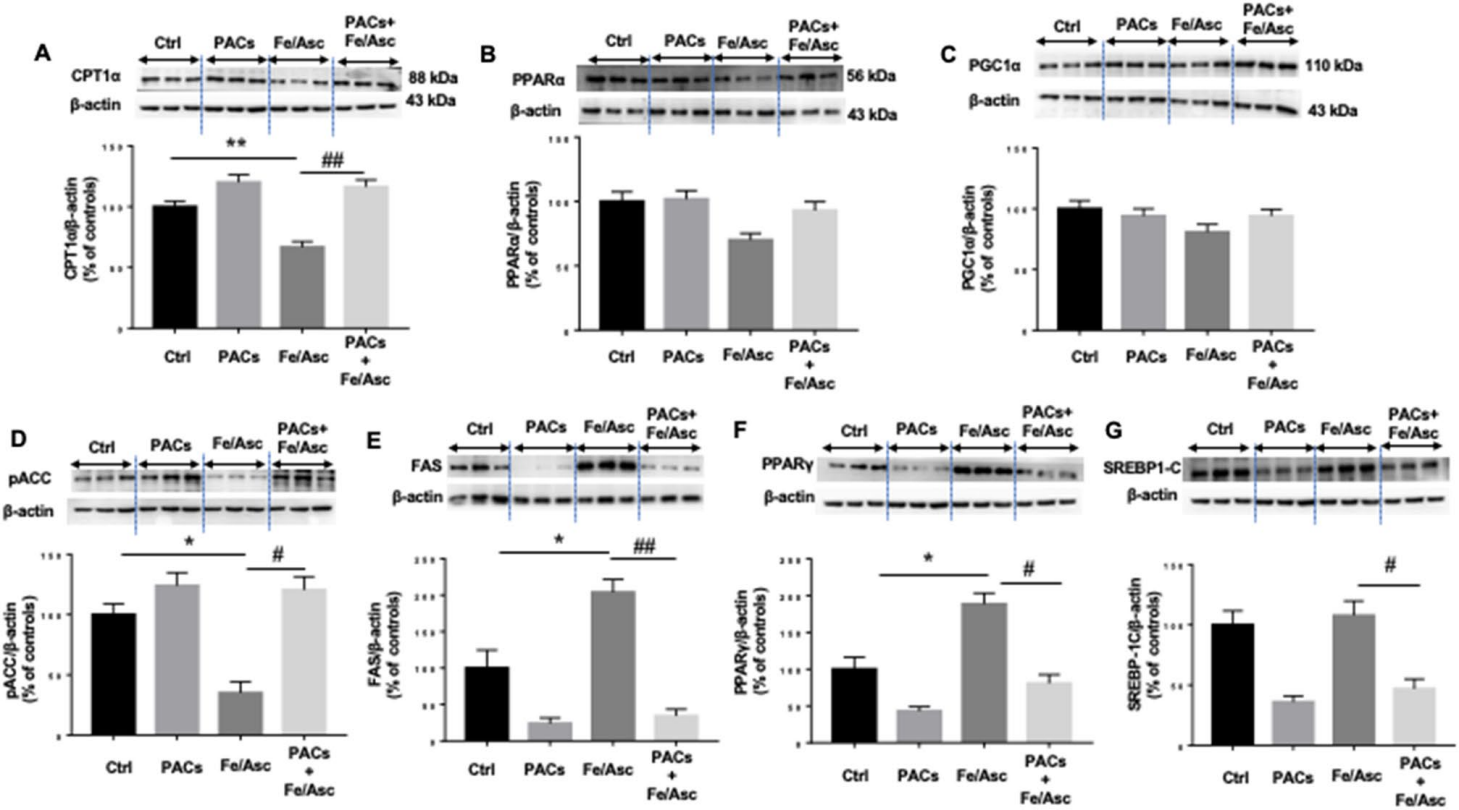
Effects of PACs on FA β-oxidation and lipogenesis in Caco-2/15 cells. Proanthocyanidins (PACs, 250 µg/mL), were added to the apical cell compartment for 24 h prior to treatment with Fe/Asc (200 µM/2 mM) for 6 h at 37 °C. The protein expression of specific markers of FA β-oxidation: ((**A**) CPT1α, (**B**) PPARα and (**C**) PGC1α as well as of lipogenesis: (**D**) ACC phosphorylation, (**E**) FAS, (**F**) PPARγ and (**G**) SREBP1) was assessed by Western-blot. Results represent the means ± SEM of 3 independent experiments, each in triplicate. A representative Western blot is shown, illustrating an experiment in triplicate on the same gel and at with same exposure. *P < 0.05; **P < 0.01 vs untreated cells (ctrl). ^#^P < 0.05; ^##^P < 0.01 vs Fe/Asc treated cells. *CPT1α* carnitine palmitoyl transferase I alpha, *PPAR* peroxisome proliferator-activated receptor, *PGC1α* peroxisome proliferator-activated receptor gamma coactivator 1-alpha, *ACC* Acetyl-CoA carboxylase, *FAS* Fatty acid synthase, *SREBP1-c* Sterol regulatory element-binding protein.

**Figure 5:**
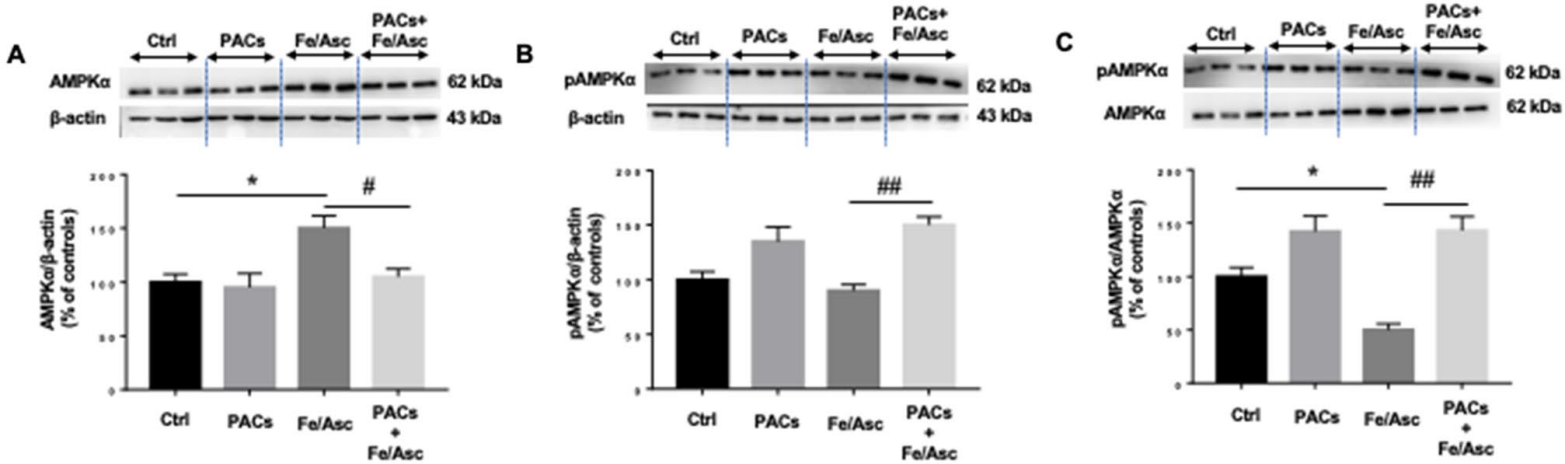
Effects of PACs on AMP-activated protein kinase alpha (AMPKα) regulating fatty acid β-oxidation and lipogenesis in Caco-2/15 cells. Proanthocyanidins (PACs, 250 µg/mL) were added to the apical cell compartment for 24 h prior to treatment with Fe/Asc (200 µM/2 mM) for 6 h at 37 °C. The protein expression of AMPKα was assessed by Western Blot. Results represent the means ± SEM of 3 independent experiments, each in triplicate. A representative Western blot is shown, illustrating an experiment in triplicate on the same gel and with the same exposure. *P < 0.05 vs untreated cells (ctrl). ^#^P < 0.05, ^##^P < 0.01 vs Fe/Asc treated cells.

The next step was to appraise the effect of PACs on lipid homeostasis by focusing on key proteins, that regulate FA β-oxidation and lipogenesis. We first examined the expression of carnitine palmitoyltransferase I (CPT-1α) because this protein is the rate-controlling enzyme of the FA β-oxidation pathway. A lower level of protein expression characterized CPT-1α in response to Fe/Asc treatment, while the pre-incubation with PACs prevented its decline compared to untreated cells (Fig. 4A). These findings prompted us to determine the potential mechanisms of action by scrutinizing the protein mass of peroxisome proliferator-activated receptor alpha (PPARα) and peroxisome proliferator-activated receptor gamma coactivator 1-alpha (PGC-1α) as they represent two transcription factors necessary for the high-level expression of mitochondrial FA β-oxidation. Western blot analysis showed a slight downregulation of PPARα without changes in PGC-1α (Fig. 4B,C). Nevertheless, the presence of PACs prevented Fe/Asc-mediated PGC-1α and PPAR-α decline. Thereafter, we turned towards the regulatory enzymes controlling the lipogenesis process. As shown in Fig. 4, an important drop of phosphorylated acetyl CoA-carboxylase (pACC) and a marked rise of fatty acid synthase (FAS) were noticed following Fe/Asc administration, indicating the activation of lipogenesis (Fig. 4D,E). These changes were probably due to the induction of the transcription factors PPARγ and sterol regulatory element-binding protein 1c (SREBP1c) in Caco-2/15 cells (Fig. 4F,G). The presence of PACs was beneficial as it reversed the pro-lipogenesis effects of Fe/Asc. Since AMP-activated protein kinase alpha (AMPKα) represents a central regulator of FA β-oxidation and lipogenesis, we investigated its protein expression, which was found to be elevated in response to Fe/Asc while normalizing in response to PACs. On the whole, most of these findings sustain a protective role of PACs in lipid homeostasis (Fig. 5).

### Impact of PACs on intracellular glucose metabolism and insulin resistance

**Figure 6:**
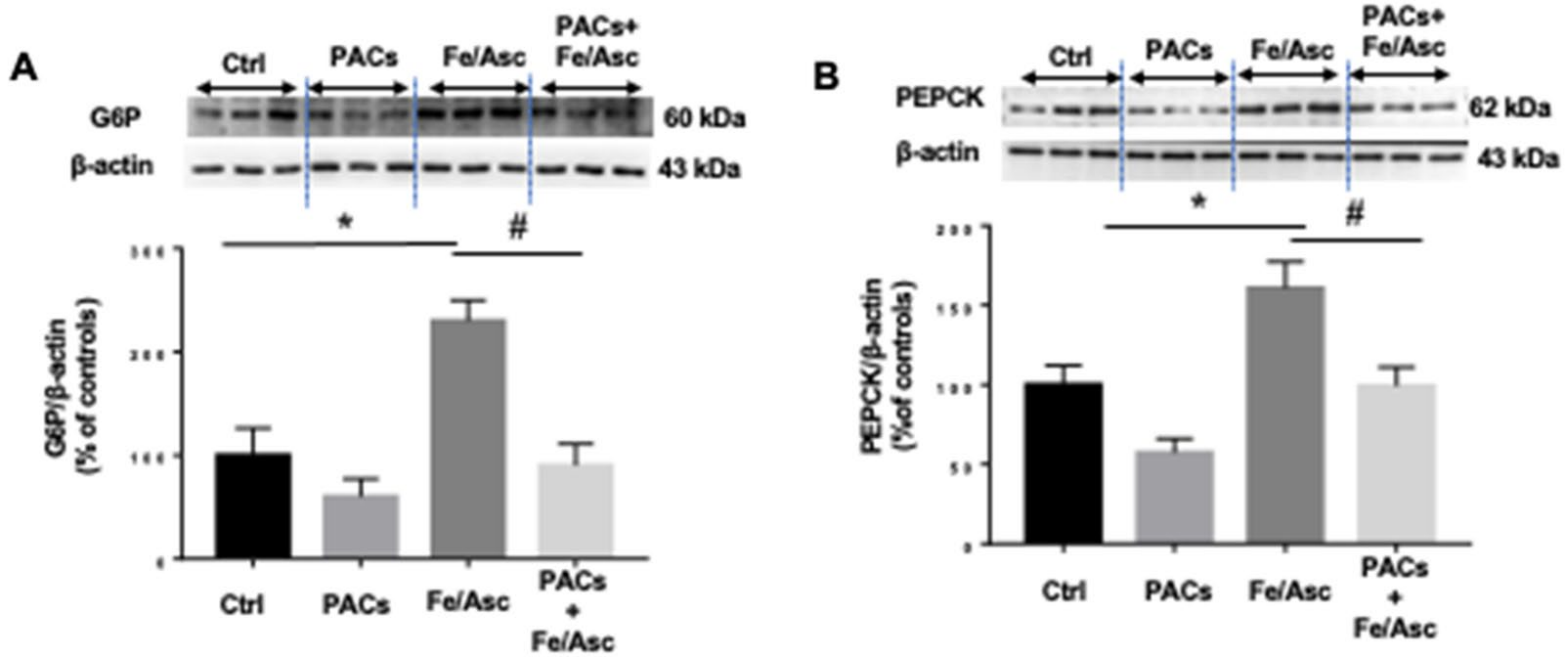
Effects of PACs on neoglucogenesis in Caco-2/15 cells. Proanthocyanidins (PACs, 250 µg/mL) were added to the apical cell compartment for 24 h prior to treatment with Fe/Asc (200 µM/2 mM) for 6 h at 37 °C. The protein expression of (**A**) G6Pase and (**B**) PEPCK was assessed by Western blot. Results represent the means ± SEM of 3 independent experiments, each in triplicate. A representative Western blot is shown, illustrating an experiment in triplicate on the same gel and at the same exposure. *P < 0.05 vs untreated cells (ctrl). ^#^P < 0.05 vs Fe/Asc treated cells. *G6Pase* glucose-6-phosphatase, *PEPCK* phosphoenol pyruvate carboxykinase.

Gluconeogenesis is strictly controlled by the activities of rate-limiting enzymes such as phosphoenolpyruvate carboxykinase (PEPCK) and glucose-6-phosphatase (G6Pase). We explored the status of these enzymes by analyzing their protein expression using Western blot. Figure 6 shows that the addition of Fe/Asc to Caco-2/15 cells raised the level of the two important enzymes, but PACs counterbalanced their Fe/Asc-induced upregulation.

**Figure 7:**
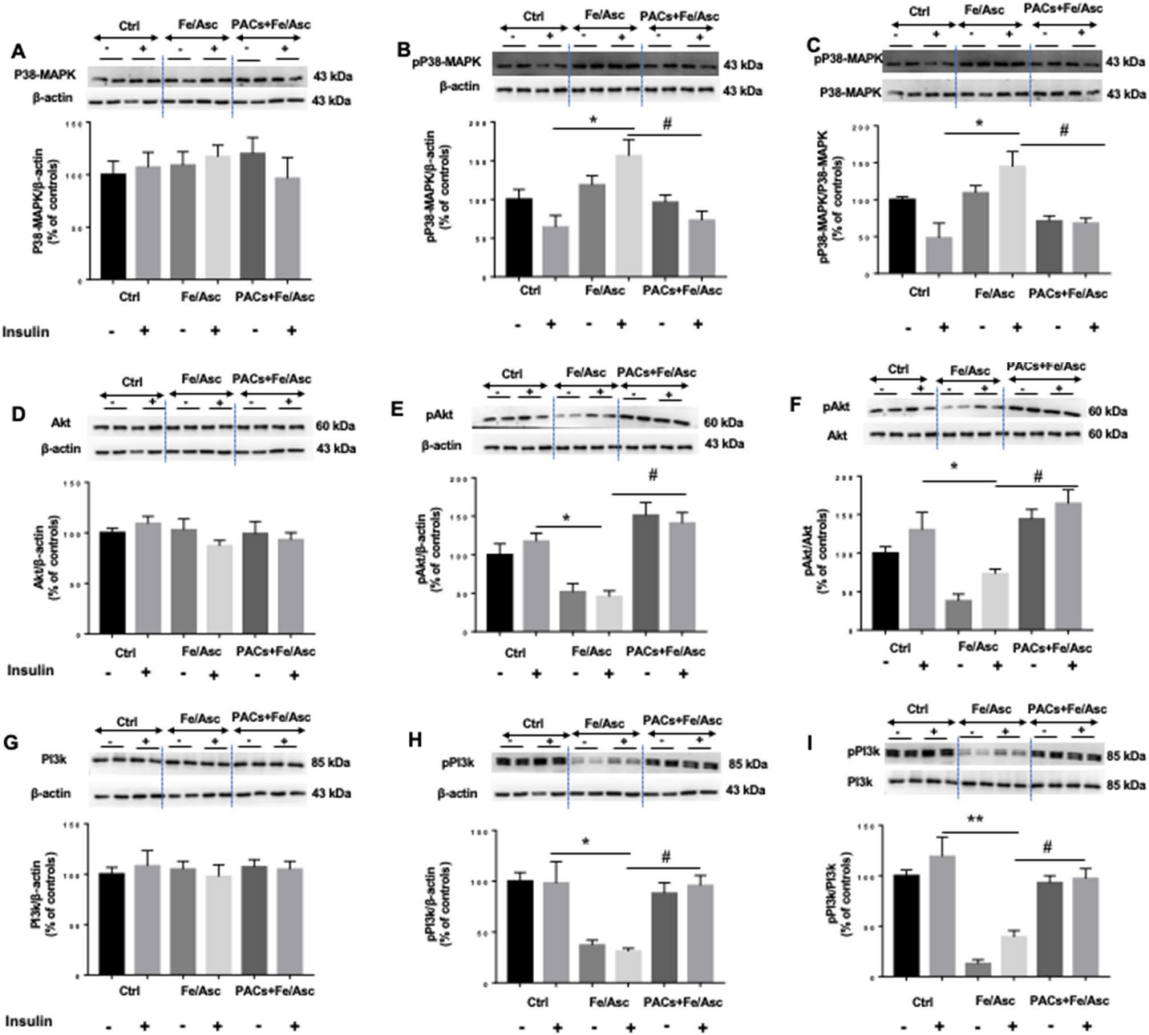
Effects of PACs on insulin sensitivity in Caco-2/15 cell line. Proanthocyanidins (PACs, 250 µg/mL) were added to the apical cell compartment for 24 h prior to treatment with Fe/Asc (200 µM/2 mM) for 6 h at 37 °C. Two hours before the end of the 6 h-incubation with Fe/Asc mixture, insulin (100 nM) was added to evaluate insulin sensitivity. The protein expression of (**A**) p38-MAPK, (**B**) phospho p38-MAPK, (**D**) Akt, (**E**) phospho Akt, (**G**) PI3k and (**H**) phospho PI3k was evaluated by Western blot. The ratios (**C**) phospho p38-MAPK/p38-MAPK, (**F**) phospho Akt/Akt and (**I**) phospho PI3k/PI3k were then calculated. Results represent the means ± SEM of 3 independent experiments each in duplicate. A representative Western blot is shown, illustrating an experiment in triplicate on the same gel and with the same exposure. *P < 0.05 vs +/− insulin untreated cells (ctrl). ^#^P < 0.05 vs Fe/Asc +/− insulin treated cells. *p38-MAPK* p38-mitogen activated protein kinase, *PI3k* phosphoinositide 3-kinase, *Akt* protein kinase B.

Since insulin signaling modulates the processes of lipogenesis, FA β-oxidation and gluconeogenesis, it was important to investigate the influence of Fe/Asc and PACs on MAPKs and PI3K/Akt, the two main insulin signaling pathway. To this end, Caco-2/15 cells were pre-incubated with insulin before the other Caco-2/15 cell treatments in order to activate the signaling pathway. While Fe/Asc raised p38-MAPK protein expression and phospho p38-MAPK/p38-MAPK ratio, while PACs prevented these actions (Fig. 7A–C). On the other hand, whereas Fe/Asc lowered the protein mass of phospho Akt (pAkt) and pAkt/Akt ratio, PACs showed an ability to maintain their normal level. A similar trend was noted for PI3K (Fig. 7D–I). Therefore, these observations are supportive of the favorable role of PACs in insulin signaling.

## Discussion

Polyphenols are well known for their pleiotropic biological and medicinal effects, which explains the attraction of many scientists and food industries for these fascinating plant-derived natural products. PACs stand out from the variety and high number of polyphenols because of their pharmacological and therapeutic impacts on chronic disorders^20,21^. Despite their praiseworthy features, there is still much that remains unknown about their influence on the GI tract, which deploys extended digestive, immunologic, neuronal and metagenomics networks^22^. For this reason, efforts have been dedicated in the present investigation to clarify the role of PACs in intestinal OxS, antioxidant defense, inflammation, FA β-oxidation, lipogenesis and gluconeogenesis, while at the same time attempting to elucidate underlying mechanisms such as insulin signaling and transcription factors status.

To probe PAC effects, we used the Caco-2/15 cell line, a popular intestinal model predominantly appreciated by the scientific community^23^, and recognized by the Food and Drug Administration for uptake and transport processes^24^. Not only do Caco-2/15 cells spontaneously differentiate into functionally and morphologically resembling human enterocytes^23,25^, but they also serve for efficient exploration of gut absorption and interactions, nutrition, toxicology, food microbiology, bioavailability tests, and screening of drug permeability in discovery program^26,27^. As in our previous reports regarding various topics^28–31^, the Caco-2/15 cell line has been instrumental in the current study to unravel the potential of PACs to regulate diverse metabolic pathways. Importantly, in this study, cells were sown on porous filters, allowing access to both sides of the bipolar intestinal epithelium: apical and basolateral compartments corresponding to the intestinal lumen or serosal circulation, respectively. Additionally, we pre-incubated Caco-2/15 cells with Fe/Asc, as this system was highly relevant in our laboratory to induce strong OxS and inflammation, which were in turn challenged by a large spectrum of effectors^17,19,32–34^. Noteworthy, Fe/Asc as a strong oxygen-radical generating system causes oxidative damage to biological macromolecules, alters intracellular redox environment and is involved in numerous pathological states^32,35,36^. Our findings show a significant elevation of lipid peroxidation (high MDA levels) along with vulnerable antioxidant defense (low GPx and SOD2 protein expressions). Furthermore, by virtue of its ability to generate lipid peroxides, the Fe/Asc complex promotes inflammation as evidenced by the increased expression of TNFα, COX2 and NF-κB. Collectively, the present results validate the radical and inflammatory generation in response to Fe/Asc.

The presence of PACs counteracted Fe/Asc-mediated lipid peroxidation. As failure of antioxidant defense could explain the induction of OxS, we examined its action on endogenous antioxidant enzymes. In fact, PACs were able to restore Fe/Asc-mediated SOD2 and GPx decline. To delineate the mechanism for the exacerbation of OxS by Fe/Asc and alleviation by PACs, we assessed the Keap1-NRF2-antioxidant response element (ARE) signalling pathway, which is considered the most powerful endogenous antioxidant regulator^37^. NRF2 is a cellular OxS sensor whose function is to maintain redox homeostasis. In normal conditions, NRF2 is continuously ubiquitinated and targeted for proteasomal degradation by its negative bound regulator Keap1^38^. In presence of endogenous and exogenous effectors, Keap1 is inactivated and downregulated, thereby disrupting the Keap1-NRF2 interaction and releasing NRF2, which translocates to the cell nucleus, binds to the promoters containing ARE, and accelerates transcription of antioxidant enzymes^39^. According to our data, the treatment of Caco-2/15 cells with PACs repressed Keap1 and likely allowed NRF2 to translocate to the nucleus allowing binding to ARE (core sequence: 5′TGACnnnGC3′) in the promoter region of antioxidant genes (SOD2 and GPx), resulting in stimulation of their transcription and coordinated up-regulation. In line with these data, the phenolic resveratrol compound prevented OxS in H_2_O_2_-treated rheumatoid arthritis fibroblast‐like synoviocytes by promoting expression of NRF2 and decreasing expression of Keap1^40^. Additionally, according to Kanzaki et al., the activation of antioxidant and cytoprotective enzymes can be associated with NRF2/Keap1 ratio^41^.

As expected from the amelioration of OxS, PACs significantly lowered inflammation as evidenced by the downregulation of TNFα and COX2. Because NF-κB is a key regulator of proinflammatory cytokines, we examined its response to PACs. In line with the trend of inflammatory agents, PACs abrogated Fe/Asc-mediated NF-κB induction and reduced NF-κB/IκB ratio. Thus, we may reasonably assume that PACs help guard against inflammation that endangers intestinal cell functions via the control of NF-κB, the master regulator of inflammatory response. It is worth mentioning that PACs may protect from inflammation by stimulating cellular antioxidant defensive mechanisms as a close relationship exists between biomarkers of redox imbalance and inflammation^42^.

The present study aimed to investigate PACs effects on intestinal lipid homeostasis. Our interesting findings showed that PACs upregulated CPT1α, on the one hand, and downregulated FAS, which represent the biomarkers of FA β-oxidation and lipogenesis, respectively. Therefore, these enzymes are potential targets of PACs, which indicates the polyphenolic potential to prevent lipid accumulation, considered as a pathological manifestation of OxS and inflammation in many tissues^43^. The molecular mechanisms are probably related to the upregulation of PPARα and downregulation of SREBP1c, two transcription factors that regulate lipid metabolism^44^. FAS is the central enzyme in lipogenesis, responsible for catalyzing the conversion of malonyl-CoA to FAs^45^. On its side, ACC catalyzes the formation of malonyl-CoA, an essential substrate for FAS. In line with our findings, *solanum nigrum* polyphenols were also found to decrease FAS and SREBPs, while increasing CPT1α and PPARα in oleic acid-treated hepatocytes^46^.

The mechanisms of actions of polyphenols are generally associated with activation of AMPKα phosphorylation^47^. Importantly, phosphorylated AMPKα in our study was lowered by Fe/Asc, but stimulated by PACs, which explains the downregulation of phospho ACC, resulting in inhibited lipogenesis and increased energy metabolism. However, the protein expression of AMPKα was increased by Fe/Asc-induced OxS in Caco2/15 cells. Accordingly, ROS and OxS may induce AMPKα via different upstream kinases^48^.

OxS and inflammation strongly induce insulin resistance and additional metabolic disorders^49,50^, which prompted us to appraise PACs capacity to regulate some key proteins relative to the insulin signaling pathway. We first focused on p38-MAPK whose expression was found upregulated by OxS while interfering with the insulin receptor signaling cascade^51^. Bothe analysis of p38-MAPK phosphorylation and calculation of phospho p38-MAPK/p38-MAPK ratio revealed a significant increase in response to Fe/Asc, which is compatible with impaired IS in Caco-2/15 cells. Validation was obtained by the decline of pAkt and PI3K. In contrast, PACs reversed the trend as they displayed the ability to lessen p38-MAPK phosphorylation and the phospho p38-MAPK/p38-MAPK ratio, while enhancing the PI3K/Akt signaling pathway, which is known to mediate the major biological actions of insulin by regulating glucose transport, protein synthesis, cellular proliferation, cell survival and gluconeogenesis^52^. The impairment in IS due to OxS and chronic inflammation can also affect gluconeogenesis process^53,54^. Analysis of the two principal enzymes involved in de novo glucose synthesis (PEPCK, G6Pase) revealed a significant increase in response to Fe/Asc whereas PACs administration brought them back to normal levels in Caco2/15 cells.

In conclusion, numerous studies in the scientific literature demonstrate significant health effects of phenolic compounds either as a specific phenolic molecule or a mixture of several polyphenols by indirect evidence. The present work points out the crucial effect of PACs (complex flavanols) in intestinal homeostasis by preventing OxS, inflammation and related metabolic disorders. Since PACs may not totally be absorbed by the small intestine, further studies are needed to investigate the mechanisms involved in their beneficial health effects in the small intestine.

## Material and methods

### Intestinal Caco-2/15 cell culture and treatment

For all the experiments, we used the human epithelial colorectal adenocarcinoma Caco-2/15 cell line, a stable clone of the parent Caco-2 cells (American Type Culture Collection, Rockville, Md., USA), that was obtained from Dr. J.F. Beaulieu (Department of Cellular Biology, Faculty of Medicine, Université de Sherbrooke, Sherbrooke, Québec, Canada). The Caco-2/15 cell line has the unique property to differentiate in vitro into polarized mature enterocytes and to form an impermeable monolayer. Caco-2/15 cells were cultured as described previously^32,34,55^ and used for the study of cell integrity^17,19^, OxS and inflammation^17,19^, and IS^56^.

### Protein expression analysis by immunoblotting

Following incubation with various treatments, Caco-2/15 cells were lysed in ice-cold buffer containing 20 mM Tris–HCl (pH 7.4), 150 mM NaCl, 1 mM Na2EDTA, 1 mM EGTA, 1% NP-40, 1% deoxycholate, 2.5 mM sodium pyrophosphate, 1 mM Na2VO4, 1 μg/mL leupeptin, and 1 mM PMSF. Protein concentration of each sample was determined using the Bradford method (Bio-Rad). Proteins were then denatured for 5 min in sample buffer containing SDS and ß-mercaptoethanol. Homogenates containing 15 μg total proteins were separated on a 10% SDS–polyacrylamide gel according to protein molecular weights and were electroblotted onto nitrocellulose membranes. Fat-free milk was used to block nonspecific sites of the membranes before adding primary antibodies overnight at 4 °C. The dilution of antibodies is reported in Supplementary Information and in our recent publication^57^. Reactive bands were captured using a ChemiDoc MP Imaging System (Bio-Rad). All data are expressed as the ratio of target protein to β-actin in the same sample.

### Statistical analysis

All values are expressed as the mean ± SEM of at least three different experiments carried out in triplicate. Data were analyzed by one-way ANOVA followed by the Tukey’s multiple comparisons test using PRISM 7.0 (GraphPad Software, San Diego, CA, USA). Differences were considered significant at *P* < 0.05.

## Supplementary Information

Supplementary Information 1.

Supplementary Information 2.
