# Supplementary material for: Intestinal protection by proanthocyanidins involves anti-oxidative and anti-inflammatory actions in association with an improvement of insulin sensitivity, lipid and glucose homeostasis
Source: Sci Rep. 2021 Feb 16;11:3878. doi: 10.1038/s41598-020-80587-5 (PMC7886900; doi:10.1038/s41598-020-80587-5)
Supplement: Supplementary file 1 — Supplementary Information 1. [file 41598_2020_80587_MOESM1_ESM.docx]

**Intestinal protection by proanthocyanidins involves anti-oxidative and anti-inflammatory actions in association with an improvement of insulin sensitivity, lipid and glucose homeostasis**

**Mireille Koudoufio**^1,2,3^**,** **Francis Feldman**^1,2,3^, **Lena Ahmarani**^1^, **Edgard Delvin**^1^**, Schohraya Spahis**^1,2,3^**, Yves Desjardins^3^ and Emile Levy** ^1,2,3 *^

^1^Research Centre, CHU Ste-Justine, ^2^Department of Nutrition, Université de Montréal, Montreal, Quebec, H3T 1C5, Canada

^3^Institute of Nutrition and Functional Foods (INAF), Université Laval, Quebec, Quebec, G1V 0A6, Canada

**Running Title**: PACs restore intestinal homeostasis

**Address for correspondence:**

Pr. Emile Levy

Research Centre

CHU Sainte-Justine

3175 Côte Ste-Catherine

Montreal, Quebec, Canada, H3T 1C5

Tel.: (514) 345-7783

E-mail:[emile.levy@recherche-ste-justine.qc.ca](mailto:emile.levy@recherche-ste-justine.qc.ca)

**SUPPLEMENTARY MATERIAL AND METHODS**

*Dilution of antibodies used in the study*

Villin (94 kDa, 1/1000, BD Biosciences); occludin (59 kDa, 1/1000, Abcam); claudin (17-26 kDa, 1/1000; Novus); TNFα (26 kDa, 1/1000, ThermoFisher scientific, Waltham, MA, USA); COX2 (70 kDa, 1/1000, Novus); NF-κB (65 kDa, 1/5000, Santa Cruz Biotechnology); NRF2 (110 kDa, 1/1000, Abcam); Keap1 (70 kDa, 1/2000, Abcam), IκB (39 kDa, 1/1000, Cell Signaling Biotechnology); pIκB^Ser-32/36^ (40 kDa, 1/1000, Cell Signalling); GPx1 (26 kDa, 1/1000, Novus Biologicals); SOD2 (21 kDa, 1/3000, Invitrogen); CPT-1α (88 kDa, 1/1000, Santa Cruz); PPARα (56 kDa, 1/1000, Cayman); PGC-1α (110 kDa, 1/1000, Abcam); pACC (260 kDa, 1/1000, Milipore); ACC (280 kDa, 1/10000, Cell Signalling); FAS (280 kDa, 1/1000, Cell Signalling); PPARγ (57 kDa, 1/2000, Cayman); SREBP-1c (125 kDa, 1/1000, Abcam); G6P (60 kDa, 1/1000, Abcam); PEPCK (62 kDa, 1/1000, Santa-Cruz); phospho-Akt and AKT (60 kDa, 1/1000, Cell signaling); p38MAPK (43 kDa, 1/1000, ThermoFisher scientific); phospho p38 MAPK (43 kDa, 1/1000, Cell signaling); PI3K (85 kDa, 1/3000, Invitrogen); phospho PI3K p85 (85 kDA, 1/500, Abcam); AMPKα and pAMPKα^Thr172^ (62 kDa, 1/1000, Cell signaling). β-actin (43 kDa; 1/1000, Sigma-Aldrich), was used as a housekeeping protein.
