# Supplementary material for: Intestinal protection by proanthocyanidins involves anti-oxidative and anti-inflammatory actions in association with an improvement of insulin sensitivity, lipid and glucose homeostasis
Source: Sci Rep. 2021 Feb 16;11:3878. doi: 10.1038/s41598-020-80587-5 (PMC7886900; doi:10.1038/s41598-020-80587-5)
Supplement: Supplementary file 2 — Supplementary Information 2. [file 41598_2020_80587_MOESM2_ESM.pdf]

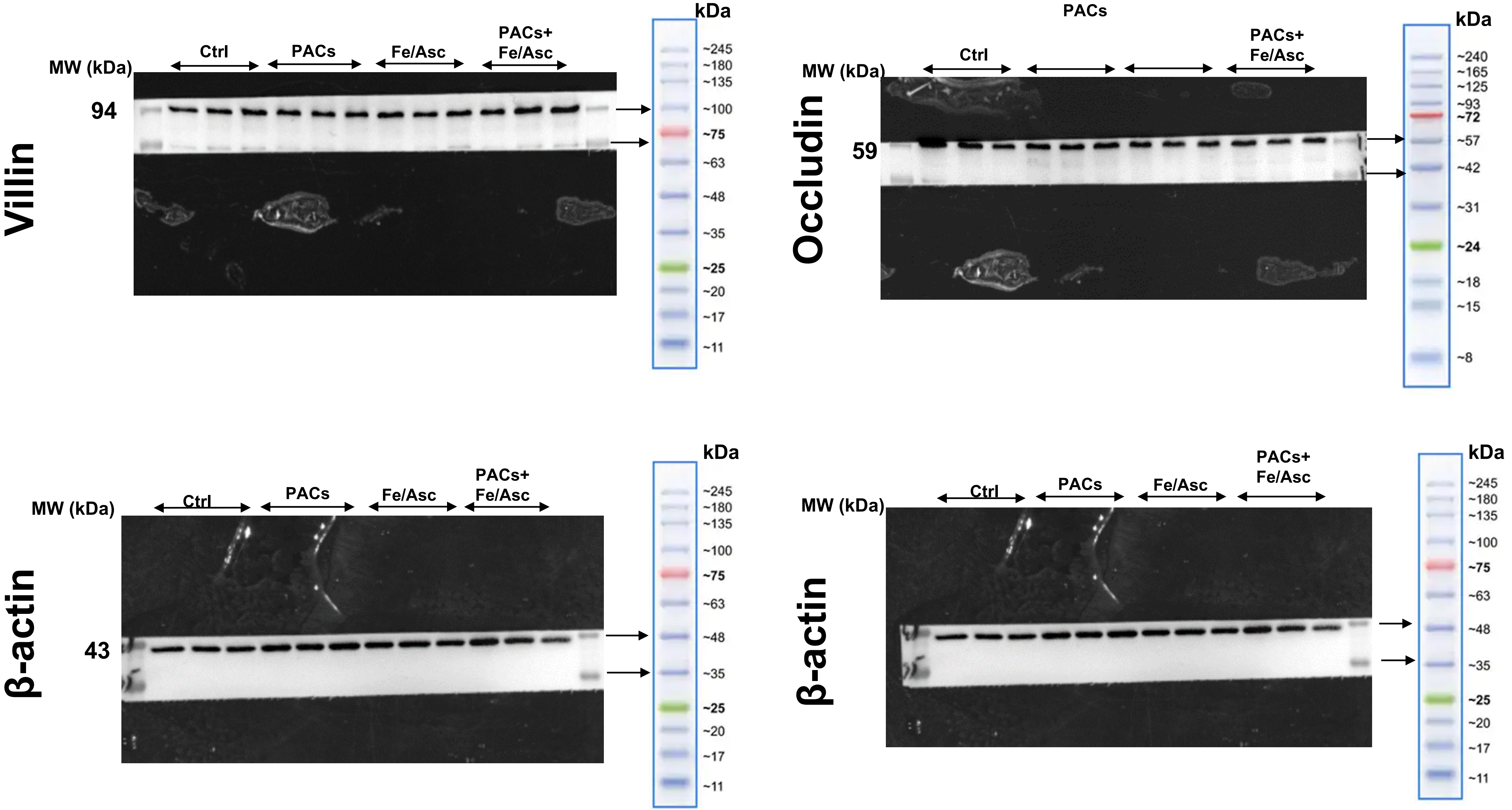
Villin
MW (kDa)
94
Ctrl
PACs
Fe/Asc
PACs+
Fe/Asc
kDa
~245
~180
~135
~100
~75
~63
~48
~35
~25
~20
~17
~11
Occludin
MW (kDa)
59
Ctrl
PACs
PACs+
Fe/Asc
kDa
~240
~165
~125
~93
~72
~57
~42
~31
~24
~18
~15
~8
β-actin
MW (kDa)
43
Ctrl
PACs
Fe/Asc
PACs+
Fe/Asc
kDa
~245
~180
~135
~100
~75
~63
~48
~35
~25
~20
~17
~11
β-actin
MW (kDa)
Ctrl
PACs
Fe/Asc
PACs+
Fe/Asc
kDa
~245
~180
~135
~100
~75
~63
~48
~35
~25
~20
~17
~11

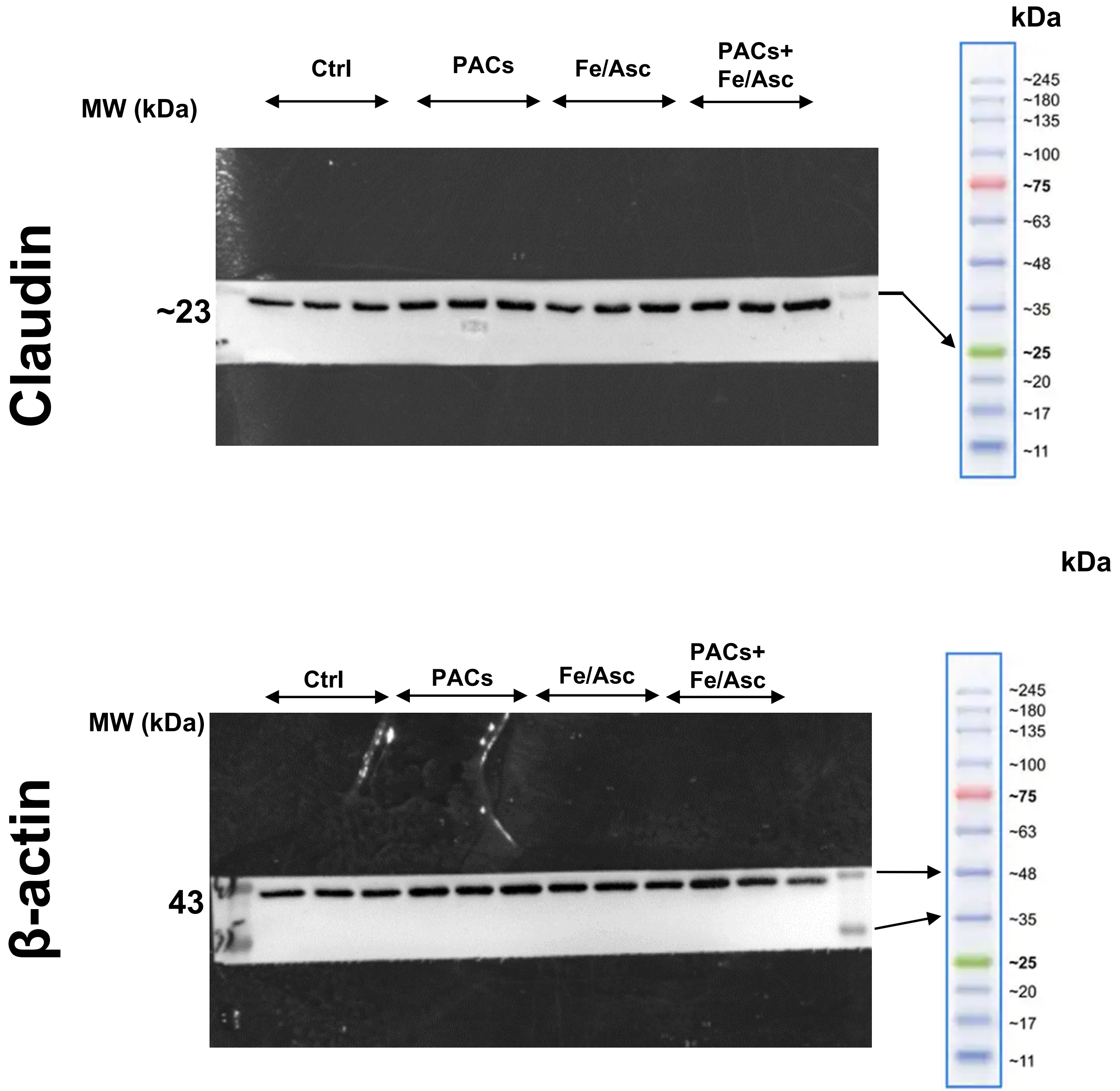
Claudin
MW (kDa)
Ctrl
PACs
Fe/Asc
PACs+
Fe/Asc
~23
kDa
~245
~180
~135
~100
~75
~63
~48
~35
~25
~20
~17
~11
β-actin
MW (kDa)
Ctrl
PACs
Fe/Asc
PACs+
Fe/Asc
43
kDa
~245
~180
~135
~100
~75
~63
~48
~35
~25
~20
~17
~11

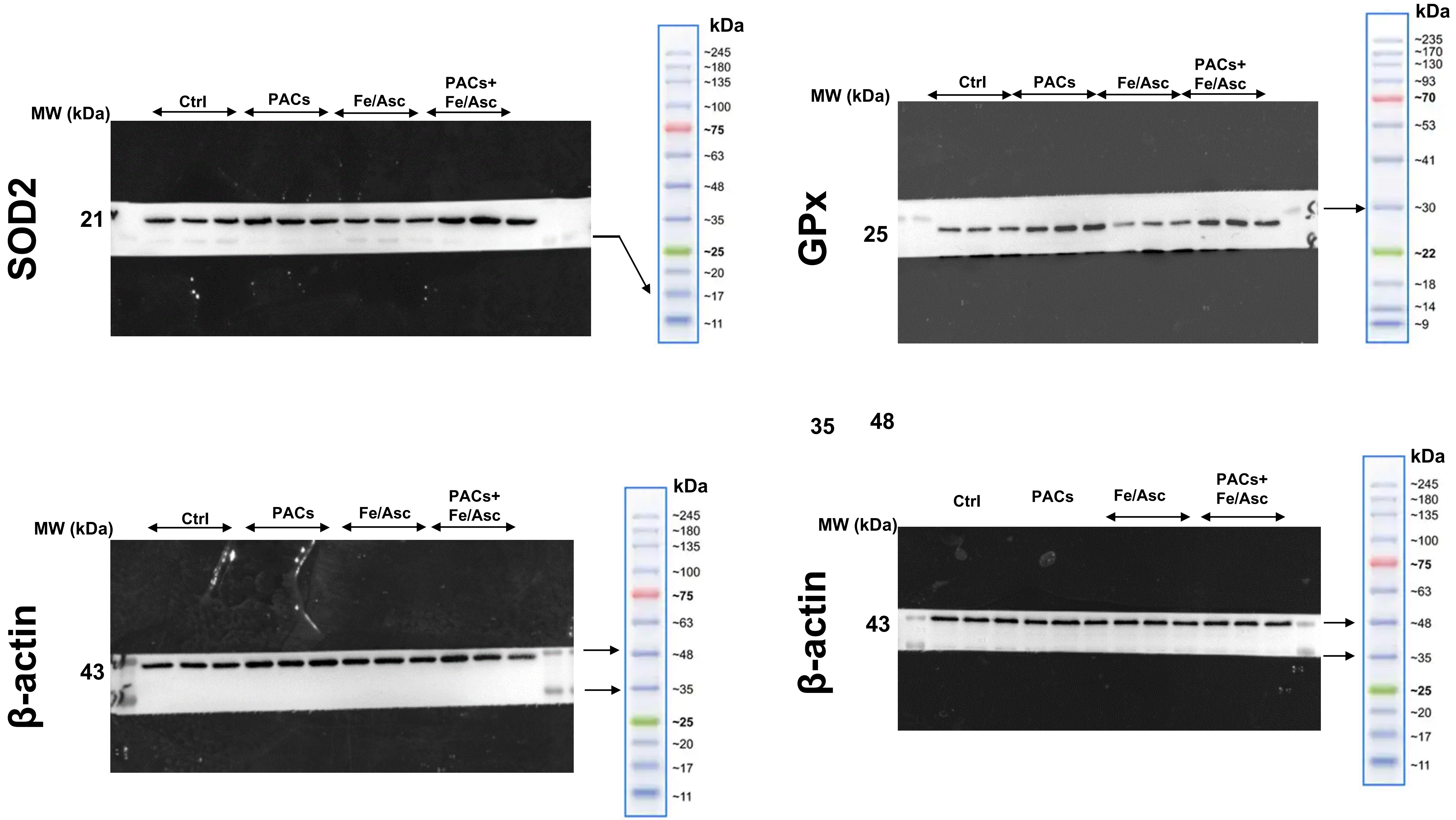

SOD2
MW (kDa)
21
Ctrl
PACs
Fe/Asc
PACs+
Fe/Asc
kDa
~245
~180
~135
~100
~75
~63
~48
~35
~25
~20
~17
~11
GPx
MW (kDa)
25
Ctrl
PACs
Fe/Asc
PACs+
Fe/Asc
kDa
~235
~170
~130
~93
~70
~53
~41
~30
~22
~18
~14
~9
35
48
β-actin
MW (kDa)
43
Ctrl
PACs
Fe/Asc
PACs+
Fe/Asc
kDa
~245
~180
~135
~100
~75
~63
~48
~35
~25
~20
~17
~11
β-actin
MW (kDa)
43
Ctrl
PACs
Fe/Asc
PACs+
Fe/Asc
kDa
~245
~180
~135
~100
~75
~63
~48
~35
~25
~20
~17
~11

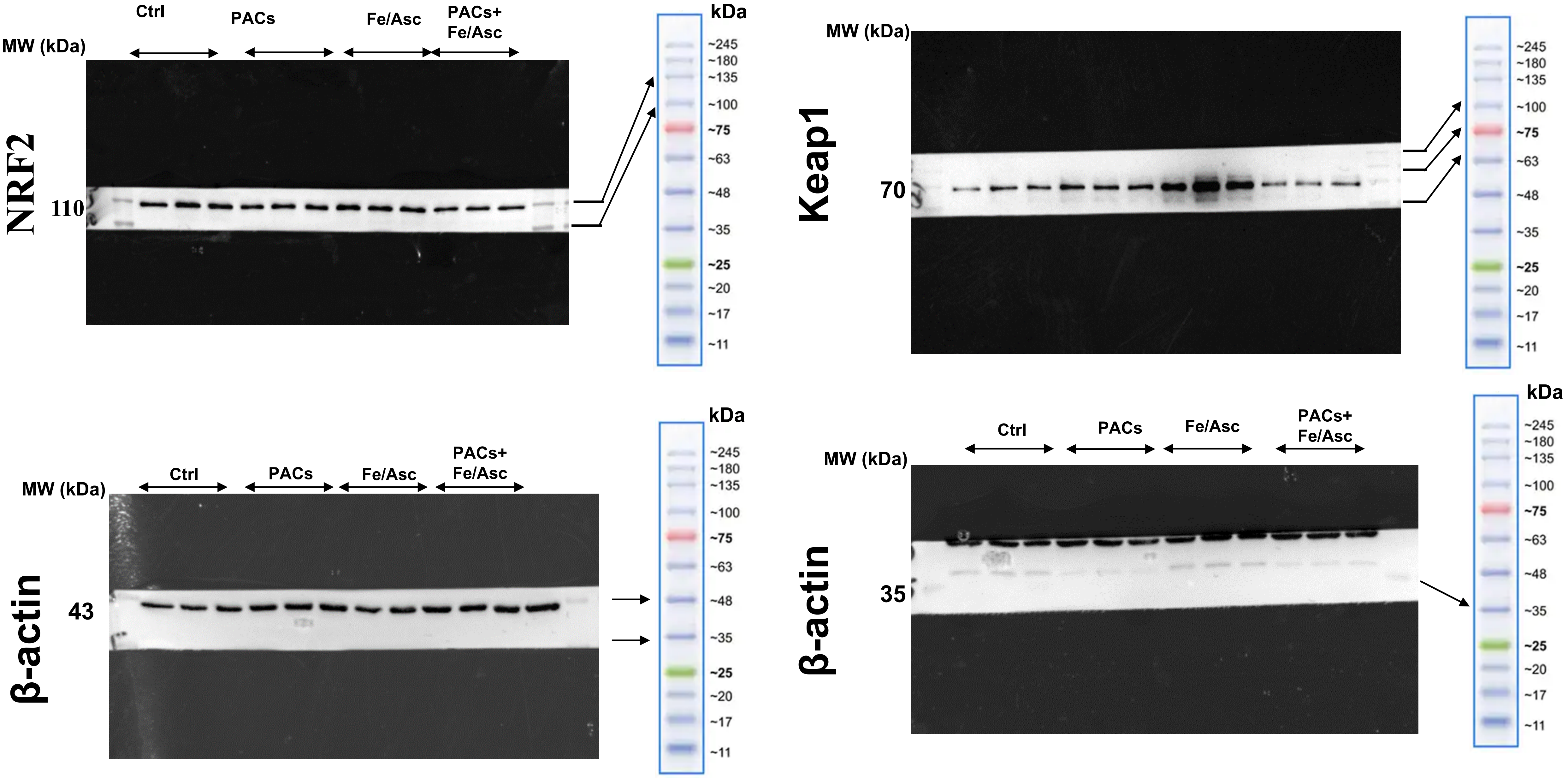

NRF2
MW (kDa)
110
Ctrl
PACs
Fe/Asc
PACs+ Fe/Asc
kDa
~245
~180
~135
~100
~75
~63
~48
~35
~25
~20
~17
~11
Keap1
MW (kDa)
70
β-actin
MW (kDa)
43
β-actin
MW (kDa)
35

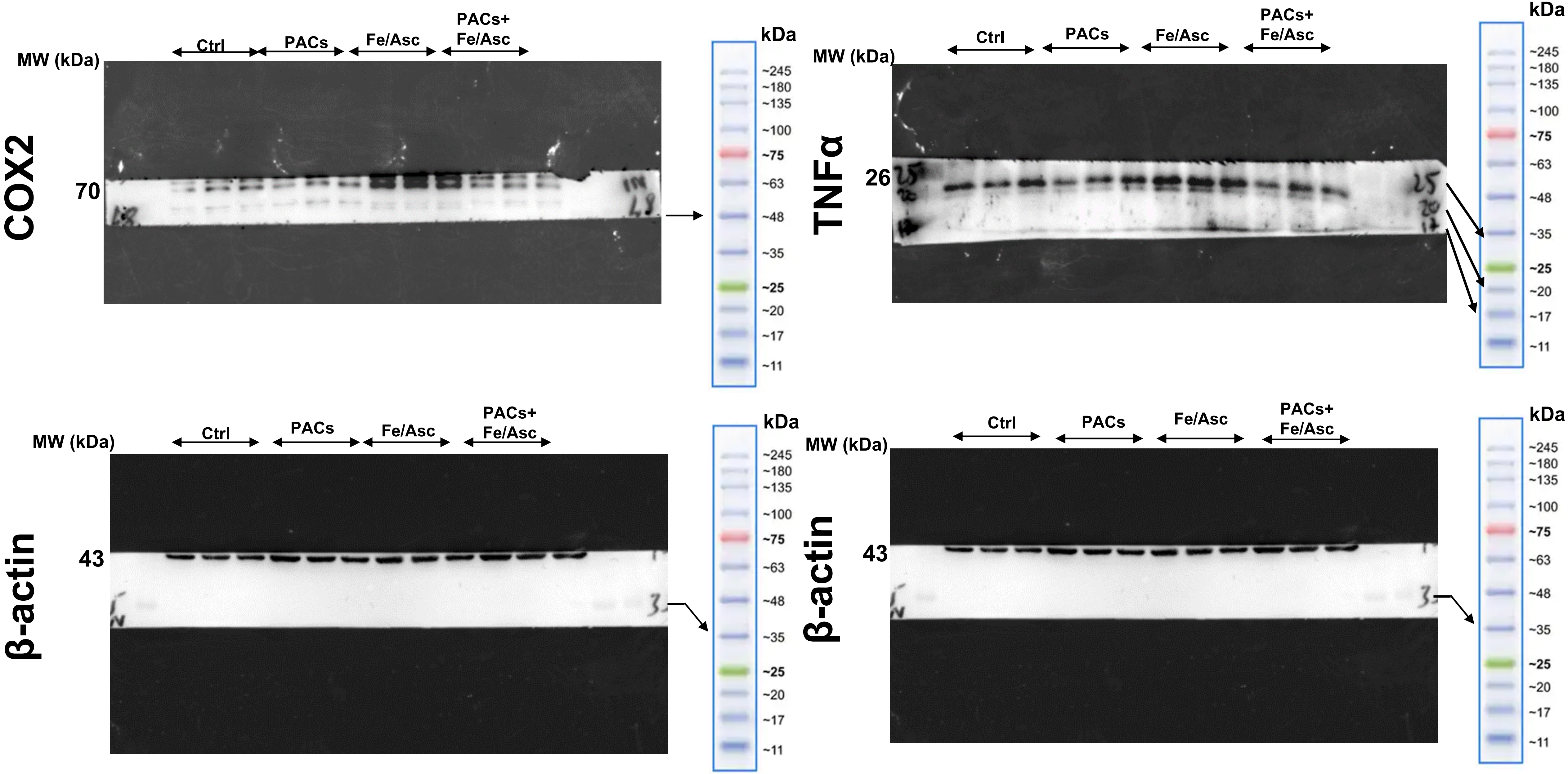
COX2
MW (kDa)
70
Ctrl
PACs
Fe/Asc
PACs+ Fe/Asc
kDa
~245
~180
~135
~100
~75
~63
~48
~35
~25
~20
~17
~11
TNFα
MW (kDa)
26
Ctrl
PACs
Fe/Asc
PACs+ Fe/Asc
kDa
~245
~180
~135
~100
~75
~63
~48
~35
~25
~20
~17
~11
β-actin
MW (kDa)
43
Ctrl
PACs
Fe/Asc
PACs+ Fe/Asc
kDa
~245
~180
~135
~100
~75
~63
~48
~35
~25
~20
~17
~11
β-actin
MW (kDa)
43
Ctrl
PACs
Fe/Asc
PACs+ Fe/Asc
kDa
~245
~180
~135
~100
~75
~63
~48
~35
~25
~20
~17
~11

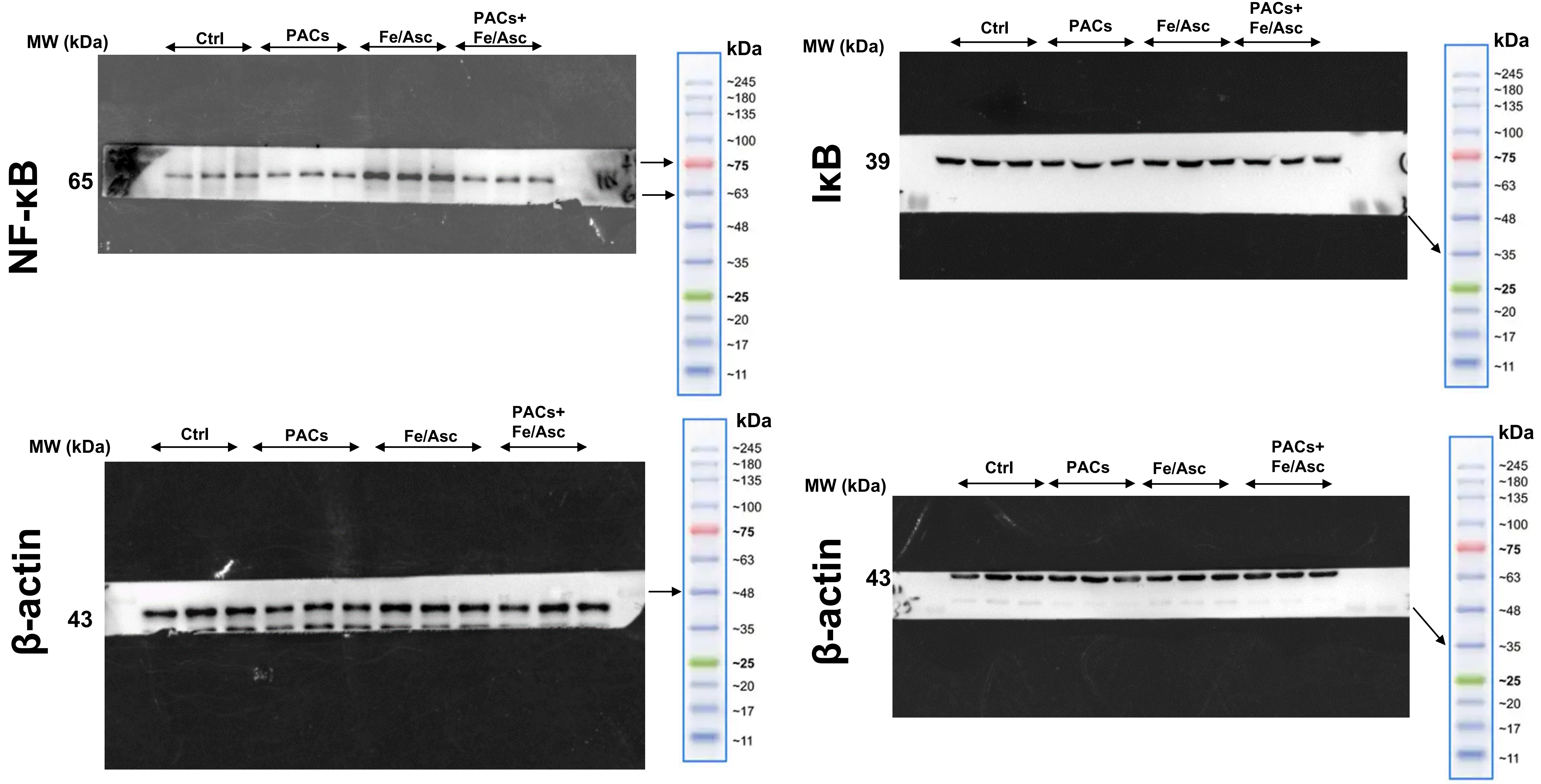

NF-κB
MW (kDa)
65
Ctrl
PACs
Fe/Asc
PACs+ Fe/Asc
kDa
~245
~180
~135
~100
~75
~63
~48
~35
~25
~20
~17
~11
IκB
MW (kDa)
39
Ctrl
PACs
Fe/Asc
PACs+ Fe/Asc
kDa
~245
~180
~135
~100
~75
~63
~48
~35
~25
~20
~17
~11
β-actin
MW (kDa)
43
Ctrl
PACs
Fe/Asc
PACs+ Fe/Asc
kDa
~245
~180
~135
~100
~75
~63
~48
~35
~25
~20
~17
~11
β-actin
MW (kDa)
43
Ctrl
PACs
Fe/Asc
PACs+ Fe/Asc
kDa
~245
~180
~135
~100
~75
~63
~48
~35
~25
~20
~17
~11

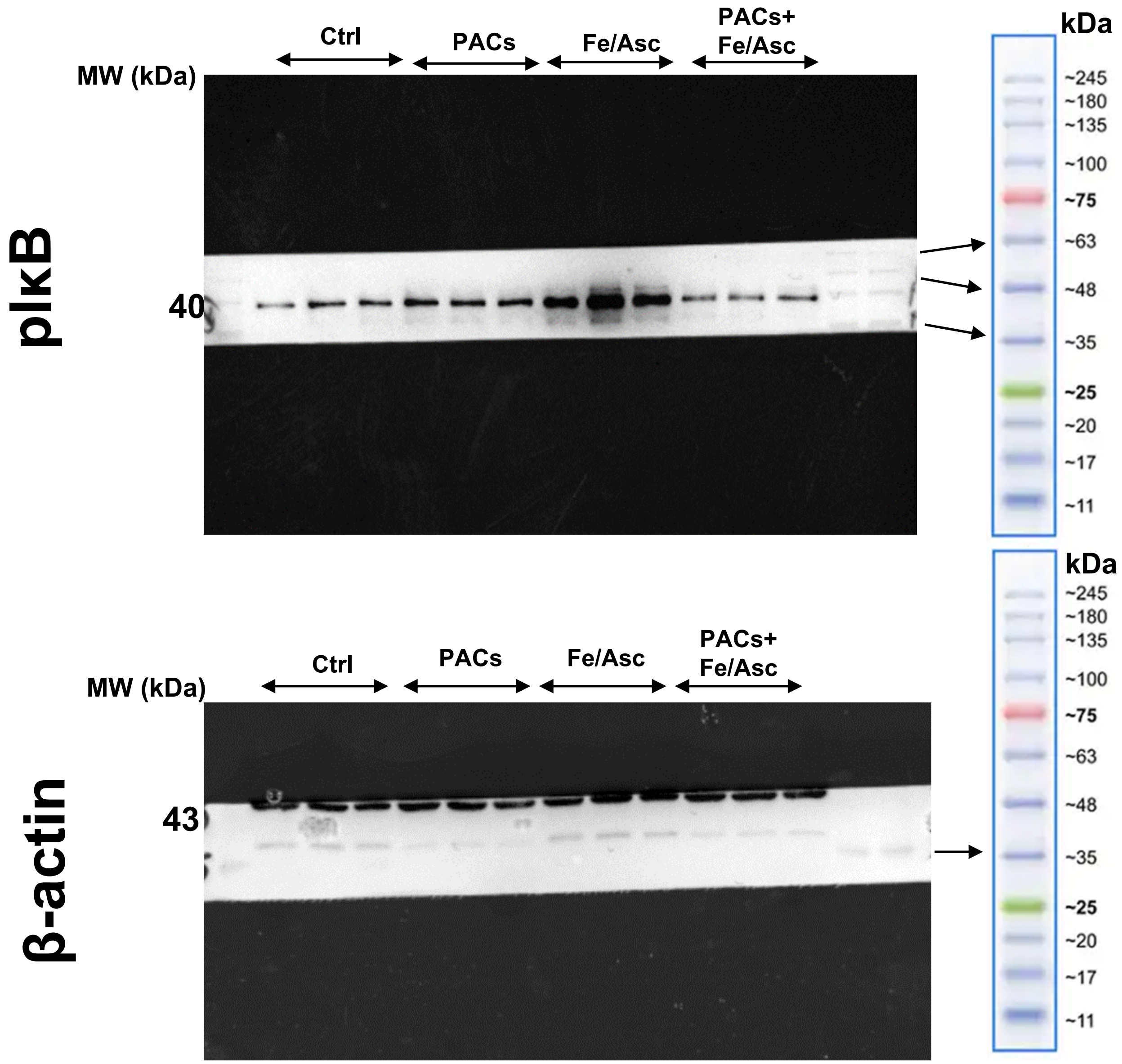
pIκB
Ctrl
PACs
Fe/Asc
PACs+
Fe/Asc
MW (kDa)
40
kDa
~245
~180
~135
~100
~75
~63
~48
~35
~25
~20
~17
~11
β-actin
Ctrl
PACs
Fe/Asc
PACs+
Fe/Asc
MW (kDa)
43
kDa
~245
~180
~135
~100
~75
~63
~48
~35
~25
~20
~17
~11

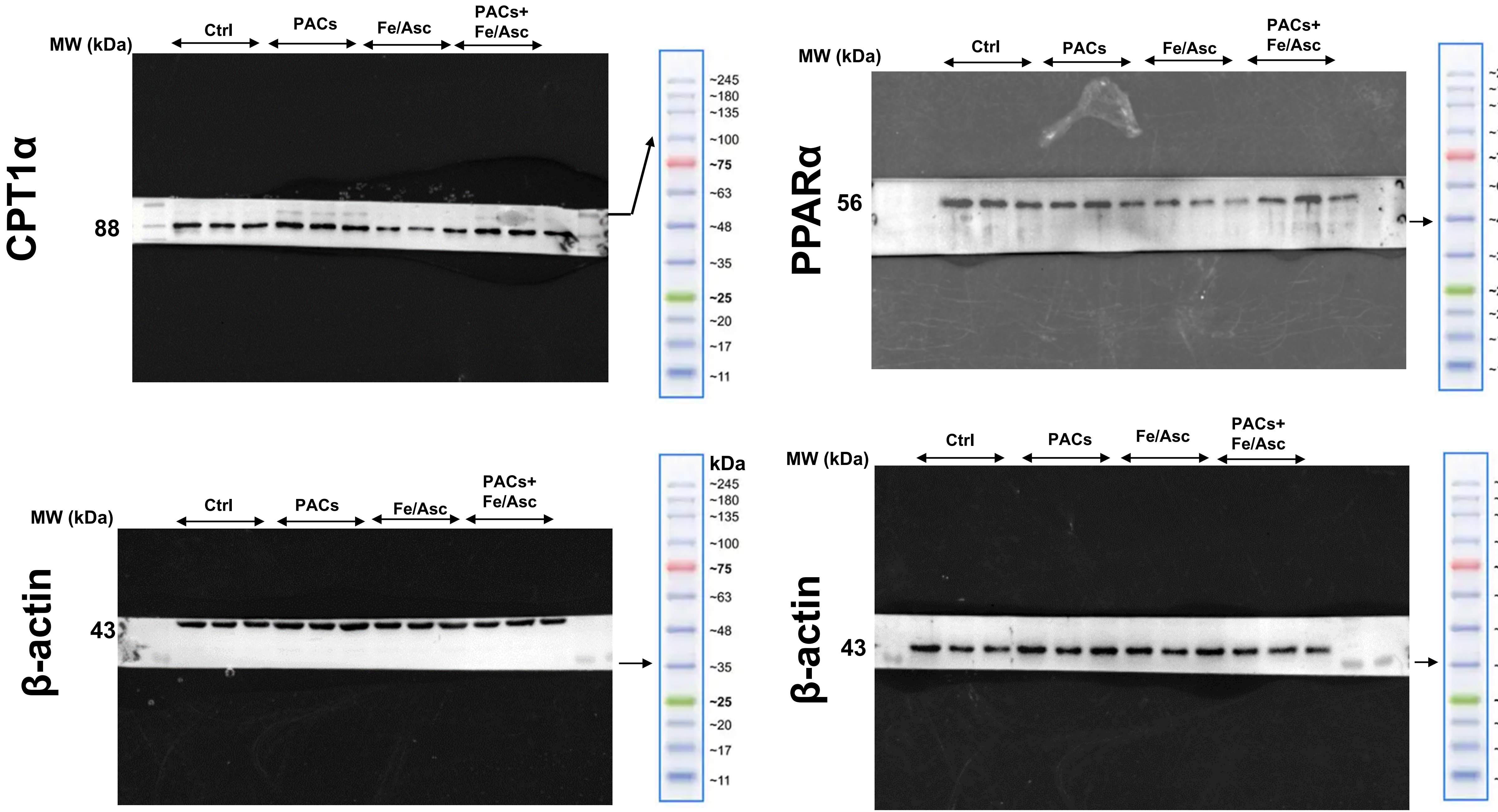

CPT1α
MW (kDa)
Ctrl
PACs
Fe/Asc
PACs+ Fe/Asc
88
~245
~180
~135
~100
~75
~63
~48
~35
~25
~20
~17
~11
PPARα
MW (kDa)
Ctrl
PACs
Fe/Asc
PACs+ Fe/Asc
56
~245
~180
~135
~100
~75
~63
~48
~35
~25
~20
~17
~11
β-actin
MW (kDa)
Ctrl
PACs
Fe/Asc
PACs+ Fe/Asc
43
kDa
~245
~180
~135
~100
~75
~63
~48
~35
~25
~20
~17
~11
β-actin
MW (kDa)
Ctrl
PACs
Fe/Asc
PACs+ Fe/Asc
43
~245
~180
~135
~100
~75
~63
~48
~35
~25
~20
~17
~11

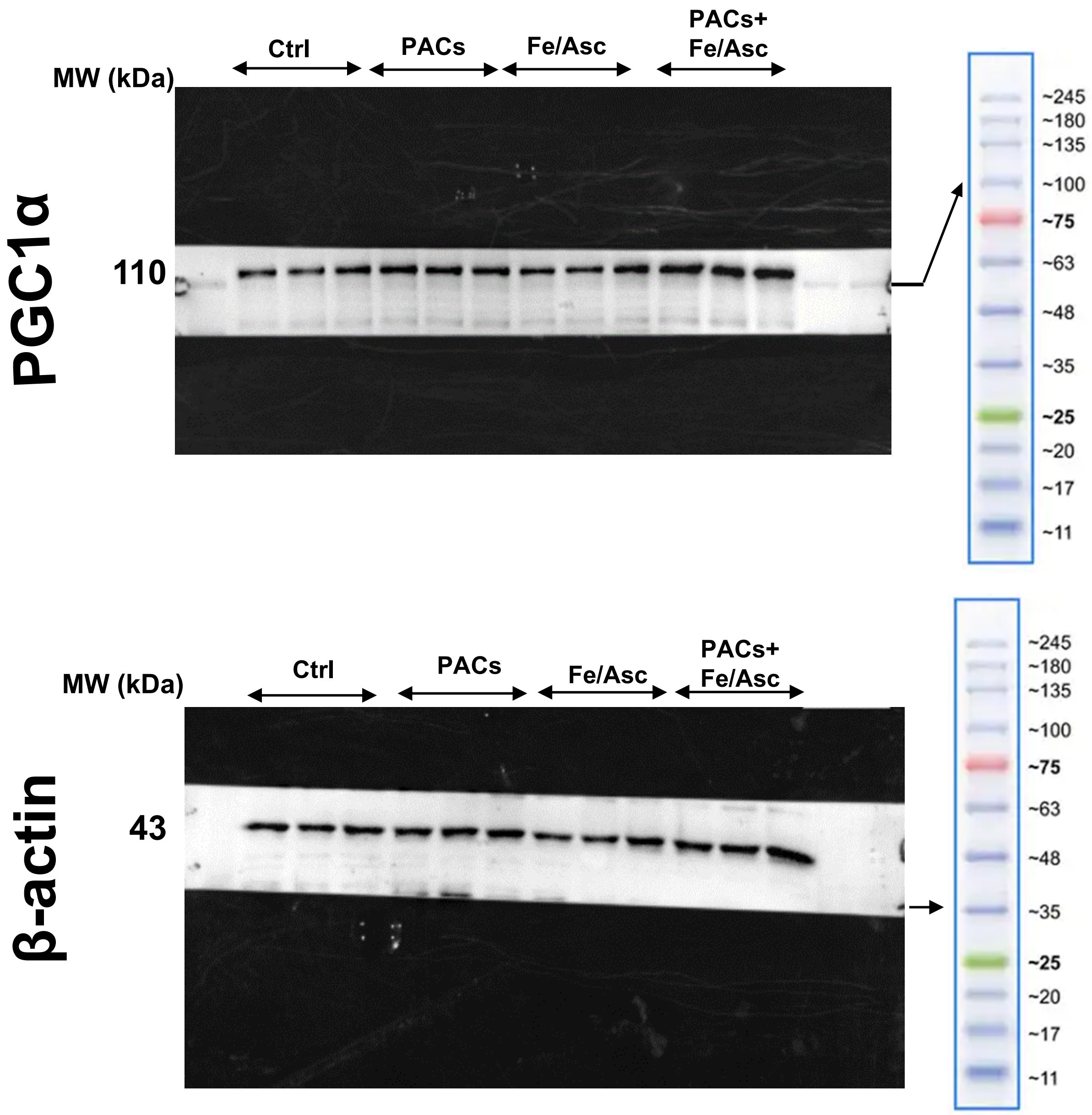

PGC1α
MW (kDa)
Ctrl
PACs
Fe/Asc
PACs+ Fe/Asc
110
~245
~180
~135
~100
~75
~63
~48
~35
~25
~20
~17
~11
β-actin
MW (kDa)
Ctrl
PACs
Fe/Asc
PACs+ Fe/Asc
43
~245
~180
~135
~100
~75
~63
~48
~35
~25
~20
~17
~11

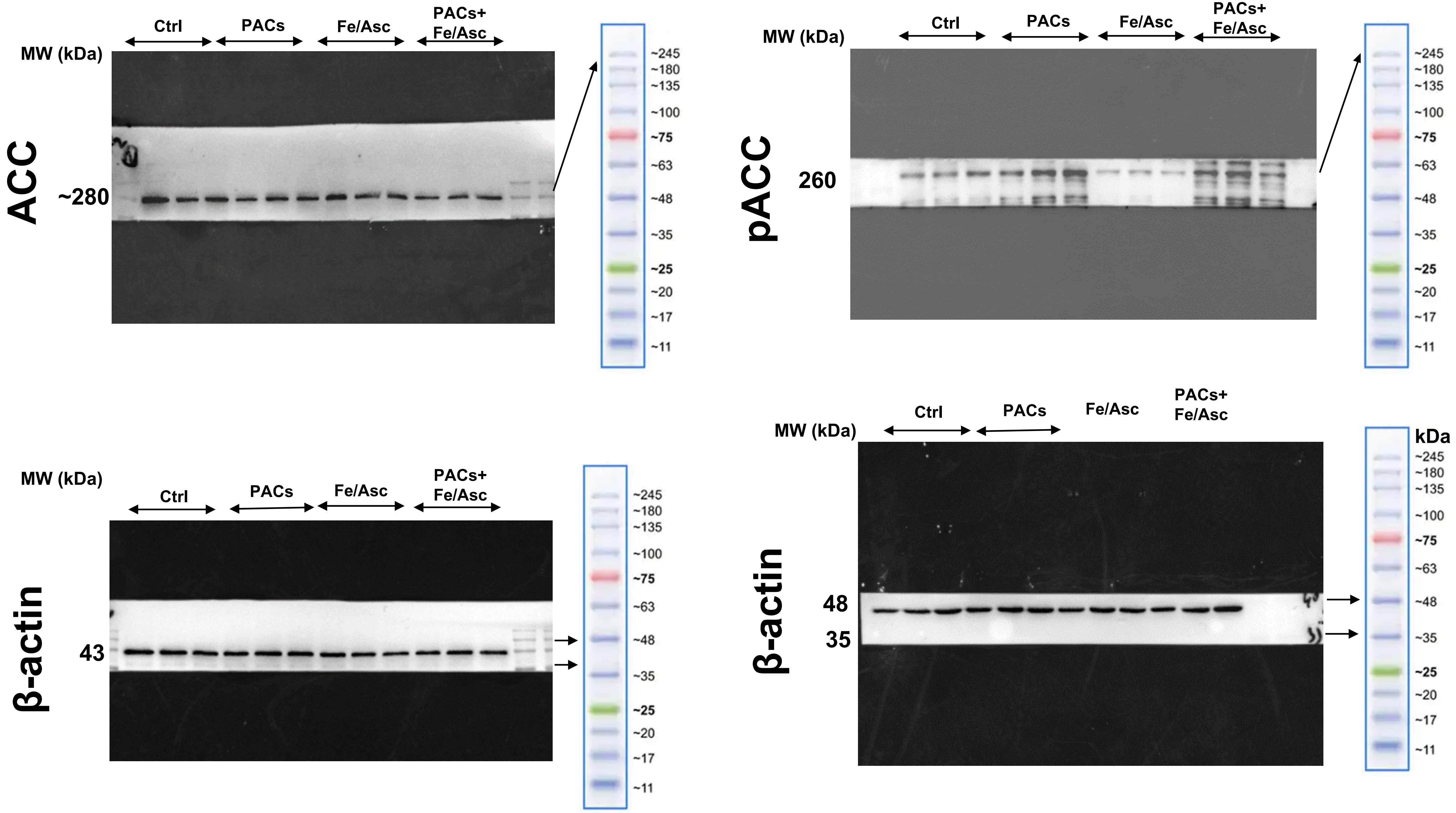
ACC
MW (kDa)
Ctrl
PACs
Fe/Asc
PACs+ Fe/Asc
~280
~245
~180
~135
~100
~75
~63
~48
~35
~25
~20
~17
~11
pACC
260
β-actin
43
48
35
kDa

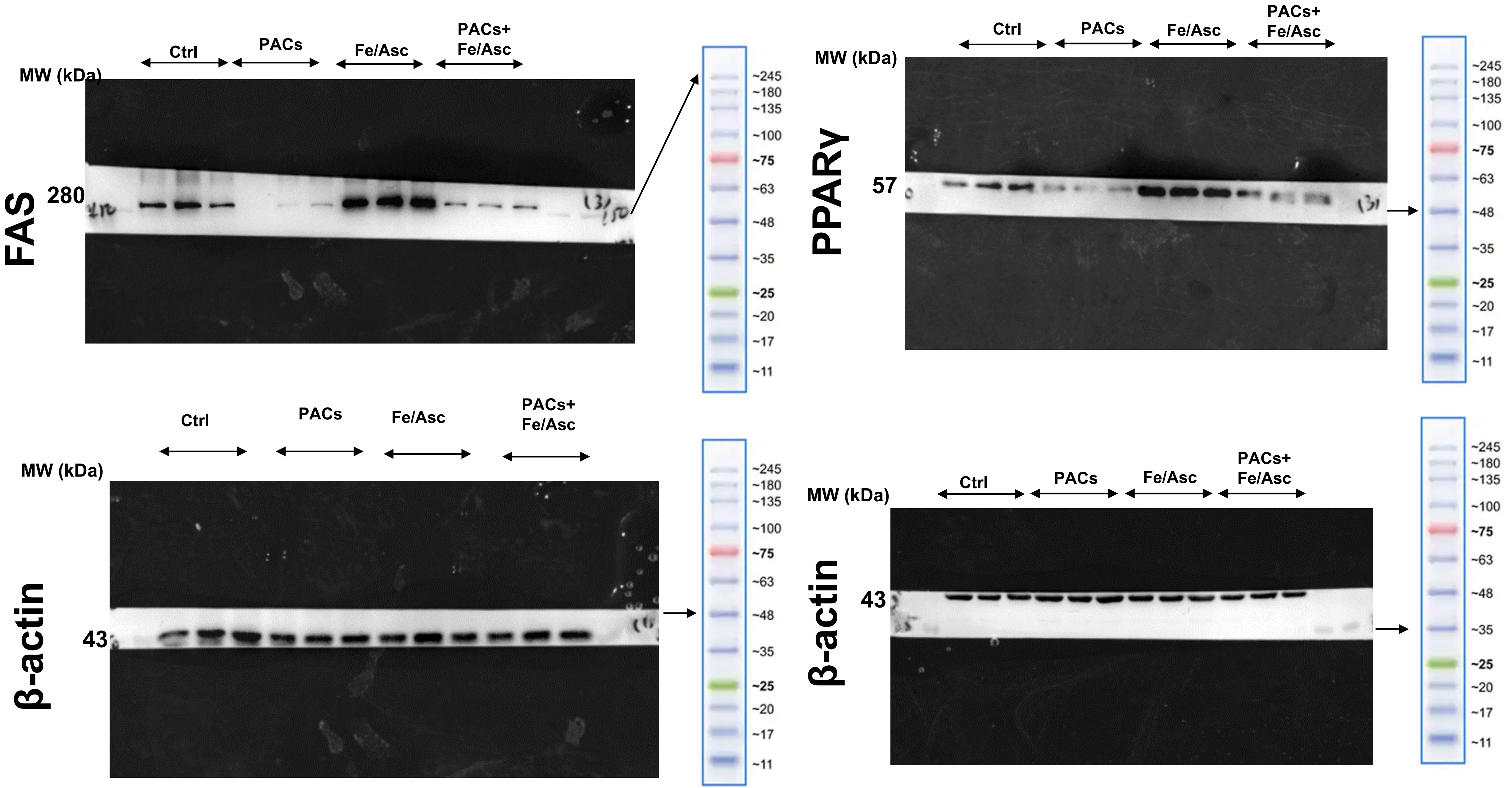
FAS
MW (kDa)
280
Ctrl
PACs
Fe/Asc
PACs+ Fe/Asc
~245
~180
~135
~100
~75
~63
~48
~35
~25
~20
~17
~11
PPARγ
57
β-actin
43

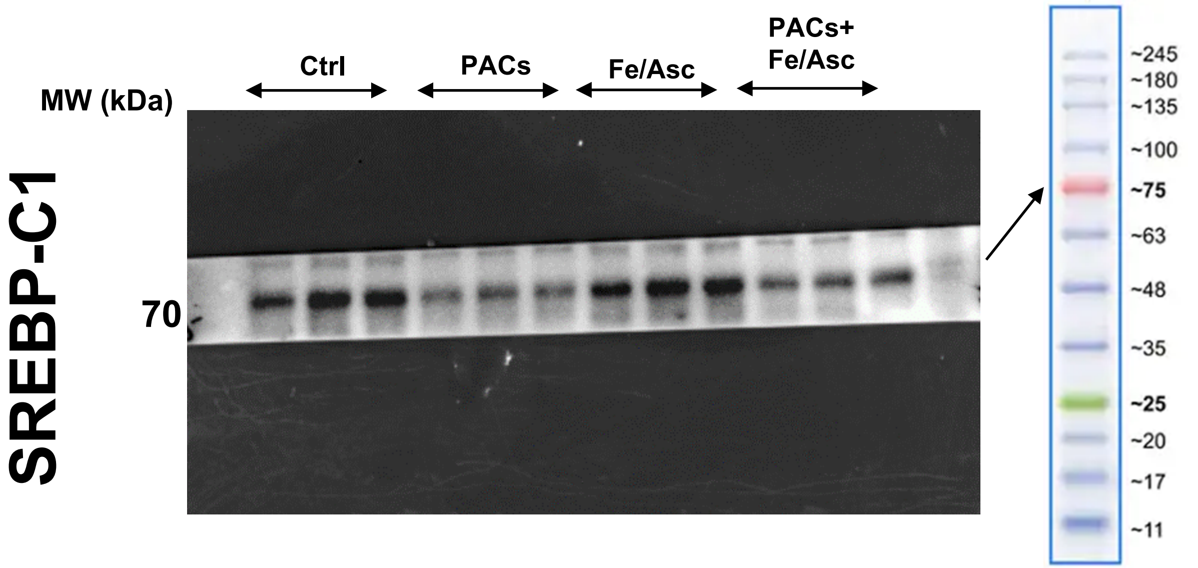
SREBP-C1
MW (kDa)
Ctrl
PACs
Fe/Asc
PACs+
Fe/Asc
70
~245
~180
~135
~100
~75
~63
~48
~35
~25
~20
~17
~11

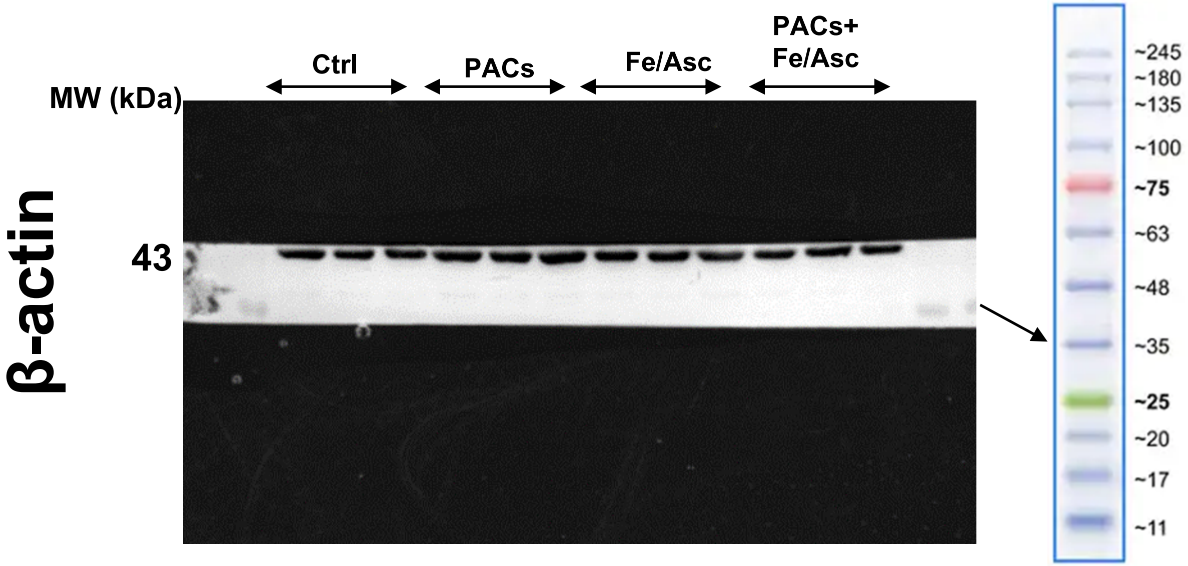
β-actin
MW (kDa)
Ctrl
PACs
Fe/Asc
PACs+
Fe/Asc
43
~245
~180
~135
~100
~75
~63
~48
~35
~25
~20
~17
~11

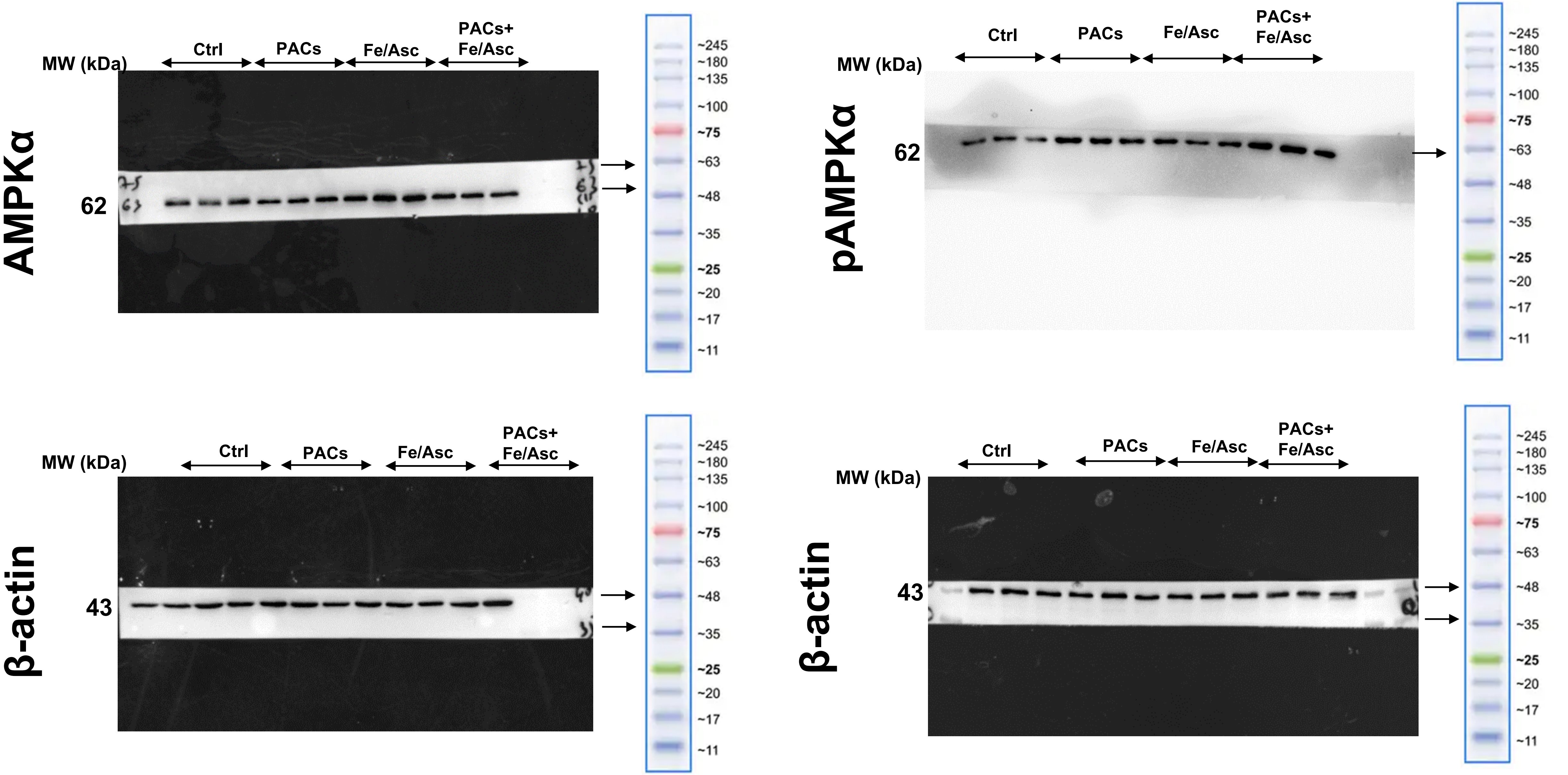
AMPKα
MW (kDa)
Ctrl
PACs
Fe/Asc
PACs+ Fe/Asc
62
~245
~180
~135
~100
~75
~63
~48
~35
~25
~20
~17
~11
pAMPKα
MW (kDa)
Ctrl
PACs
Fe/Asc
PACs+ Fe/Asc
62
β-actin
MW (kDa)
Ctrl
PACs
Fe/Asc
PACs+ Fe/Asc
43
β-actin
MW (kDa)
Ctrl
PACs
Fe/Asc
PACs+ Fe/Asc
43

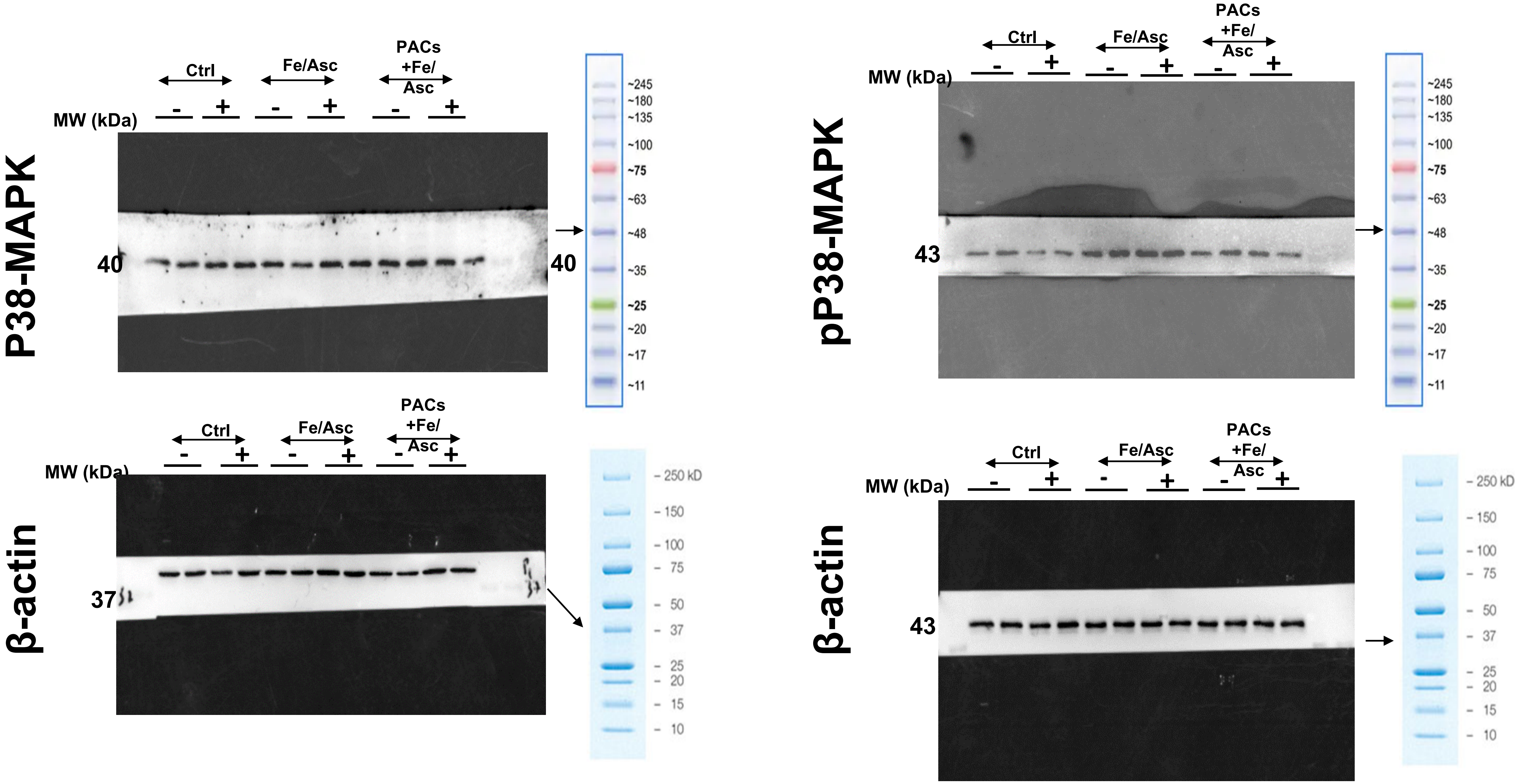

P38-MAPK
Ctrl
Fe/Asc
PACs +Fe/ Asc
MW (kDa)
- + - + - +
40
40
~245
~180
~135
~100
~75
~63
~48
~35
~25
~20
~17
~11
pP38-MAPK
Ctrl
Fe/Asc
PACs +Fe/ Asc
MW (kDa)
- + - + - +
43
~245
~180
~135
~100
~75
~63
~48
~35
~25
~20
~17
~11
β-actin
Ctrl
Fe/Asc
PACs +Fe/ Asc
MW (kDa)
- + - + - +
37
250 kD
150
100
75
50
37
25
20
15
10
β-actin
Ctrl
Fe/Asc
PACs +Fe/ Asc
MW (kDa)
- + - + - +
43
250 kD
150
100
75
50
37
25
20
15
10

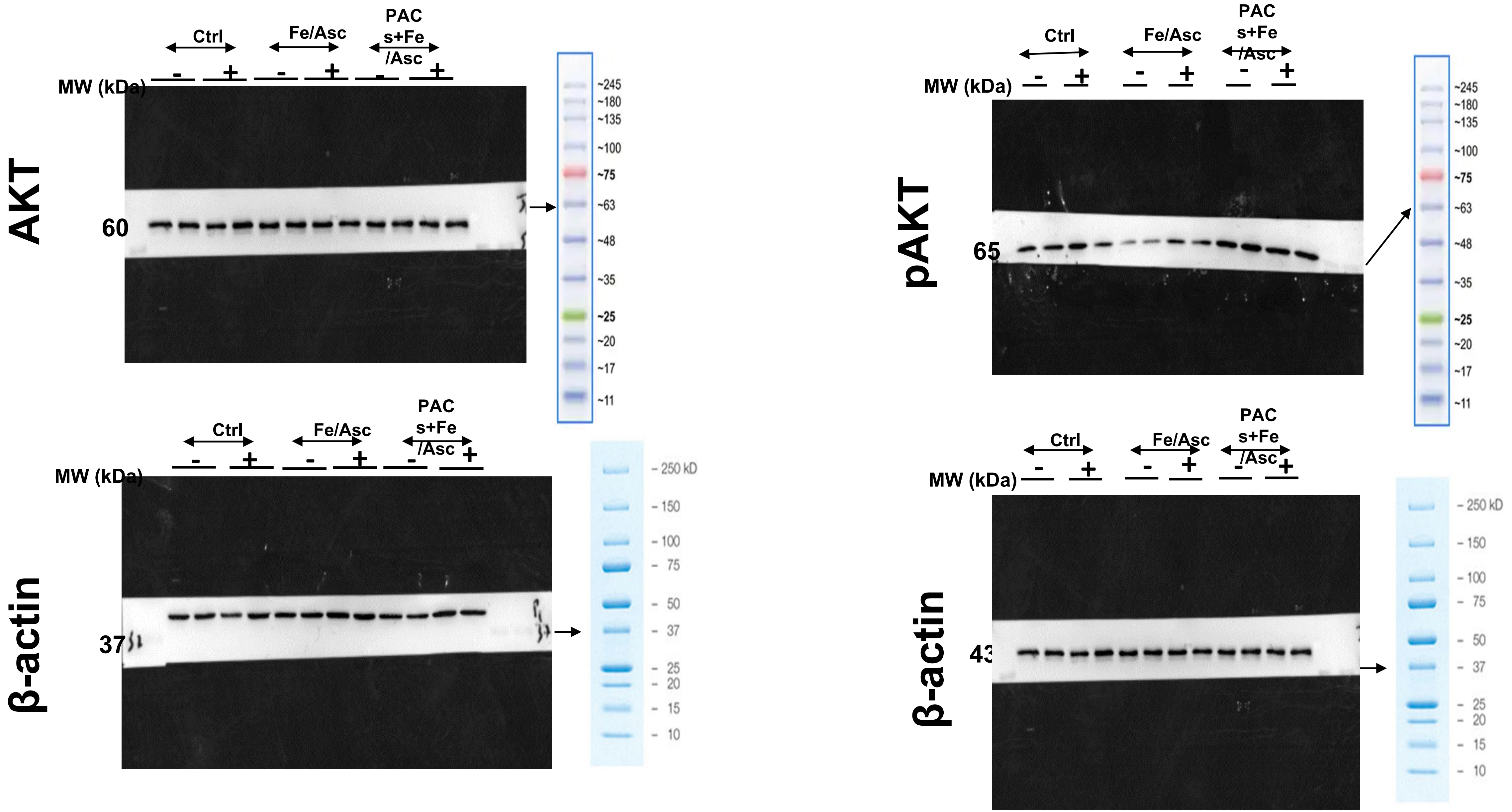
AKT
Ctrl
Fe/Asc
PAC s+Fe /Asc
MW (kDa)
- + - + - +
60
~245
~180
~135
~100
~75
~63
~48
~35
~25
~20
~17
~11
pAKT
65
β-actin
37
- 250 kD
- 150
- 100
- 75
- 50
- 37
- 25
- 20
- 15
- 10
43

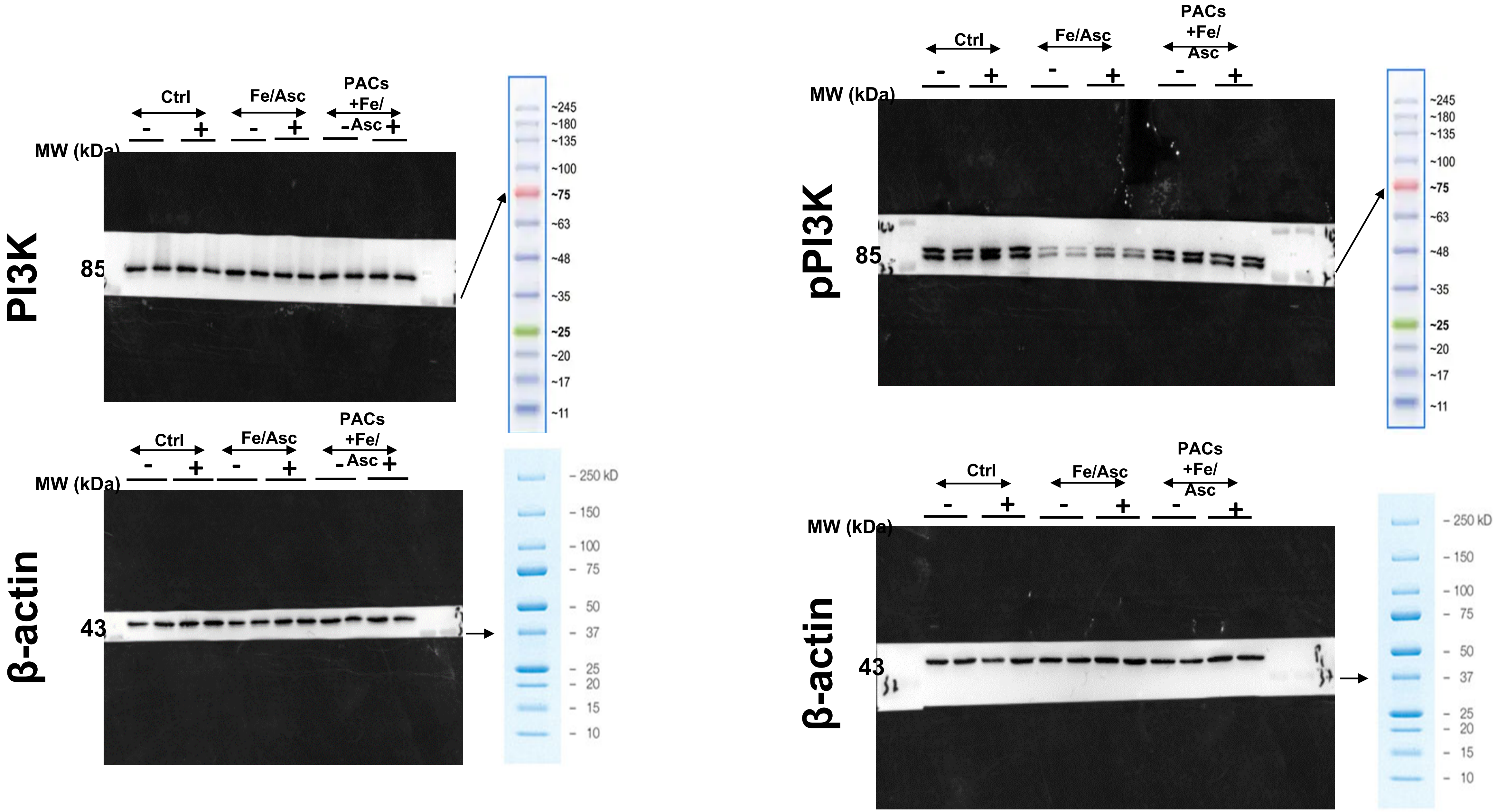

PI3K
Ctrl
Fe/Asc
PACs
+Fe/
Asc
- + - + - +
MW (kDa)
85
~245
~180
~135
~100
~75
~63
~48
~35
~25
~20
~17
~11
β-actin
43
250 kD
150
100
75
50
37
25
20
15
10
pPI3K

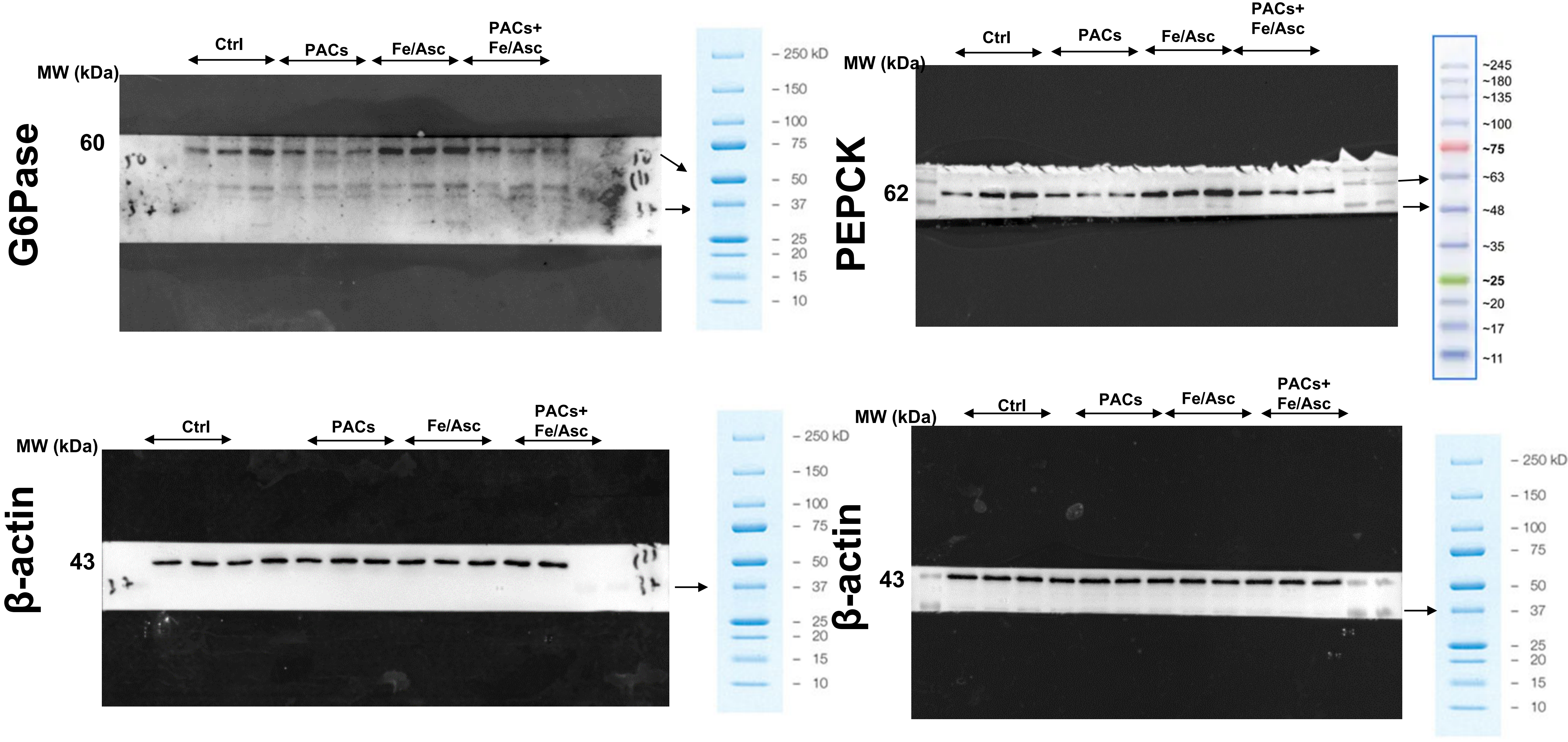

G6Pase
MW (kDa)
60
Ctrl
PACs
Fe/Asc
PACs+
Fe/Asc
250 kD
150
100
75
50
37
25
20
15
10
PEPCK
MW (kDa)
62
Ctrl
PACs
Fe/Asc
PACs+
Fe/Asc
~245
~180
~135
~100
~75
~63
~48
~35
~25
~20
~17
~11
β-actin
MW (kDa)
43
Ctrl
PACs
Fe/Asc
PACs+
Fe/Asc
250 kD
150
100
75
50
37
25
20
15
10
β-actin
MW (kDa)
43
Ctrl
PACs
Fe/Asc
PACs+
Fe/Asc
250 kD
150
100
75
50
37
25
20
15
10
